# Fibroblasts in metastatic lymph nodes confer cisplatin resistance to ESCC tumor cells via PI16

**DOI:** 10.1038/s41389-023-00495-x

**Published:** 2023-11-01

**Authors:** Lily Liang, Xu Zhang, Xiaodong Su, Tingting Zeng, Daqin Suo, Jingping Yun, Xin Wang, Xin-Yuan Guan, Yan Li

**Affiliations:** 1 State Key Laboratory of Oncology in South China, Guangdong Provincial Clinical Research Center for Cancer, Sun Yat-sen University Cancer Center, Guangzhou, China; 2 Guangdong Esophageal Cancer Institute, Department of Thoracic Oncology, Sun Yat-sen University Cancer Center, Guangzhou, China; 3 Department of Clinical Oncology, The University of Hong Kong, Hong Kong, China; 4 Guangdong-Hongkong Joint Laboratory for RNA medicine, Sun Yat-sen University, Guangzhou, China

**Keywords:** Cancer microenvironment, Oesophageal cancer, Cell death

## Abstract

Although many studies have compared tumor fibroblasts (T-Fbs) and nontumor fibroblasts (N-Fbs), less is understood about the stromal contribution of metastatic lymph node fibroblasts (LN-Fbs) to the evolving microenvironment. Here, we explored the characteristics of LN-Fbs in esophageal squamous cell carcinoma (ESCC) and the interactions between fibroblasts and ESCC tumor cells in metastatic lymph nodes. Fibroblasts were isolated from tumor, nontumor and metastatic lymph node tissues from different patients with ESCC. Transcriptome sequencing was performed on the fibroblasts. Tumor growth and drug-resistance assays were carried out, and characteristics of T-Fbs, N-Fbs and LN-Fbs were determined. Liquid chromatography-tandem mass spectrometry (LC-MS/MS) was used to assay the culture medium of fibroblasts. The results demonstrated that fibroblasts derived from different tissues had different characteristics. Coculture with LN-Fbs conditioned medium inhibited ESCC tumor cell growth and induced chemoresistance in ESCC cells. LN-Fbs induced chemoresistance to cisplatin in ESCC cells by secreting PI16. Coculture with LN-Fbs conditioned medium decreased cisplatin-induced apoptosis in ESCC cells by regulating the p38 and JNK cell signaling pathways. Survival analyses showed that patients with high PI16 expression in Fbs of lymph nodes exhibited worse overall survival. We also examined PI16 expression in interstitial tissues in ESCC tumor samples of patients receiving platinum-based therapy postsurgery and found that high PI16 expression in tumor interstitial tissues was an independent prognostic factor for ESCC patients. In addition, an in vivo assay demonstrated that PI16 knockdown increased the sensitivity of ESCC cells to cisplatin. Our results suggest that fibroblasts in metastatic lymph nodes decrease apoptosis of ESCC cells via PI16, thereby providing a cisplatin-resistance niche and supporting ESCC tumor cells to survive in metastatic lymph nodes. PI16 is also a potential target for effectively blocking the chemoresistance niche signaling circuit in response to cisplatin.

## Introduction

According to the World Cancer Research Fund international, esophageal cancer is the 7th most common cancer in men and the 13th most common cancer in women worldwide [1]. Eastern Asia exhibits the highest regional incidence rates followed by Southern Africa, Eastern Africa, Northern Europe and South Central Asia [1]. Most esophageal cancers are esophageal squamous cell carcinoma (ESCC) and it accounts for approximately 90% of esophageal cancers.

Lymph node metastasis is predictive of poor prognosis in many cancers. The frequency of lymph node metastasis is high in the early stage of esophageal cancer. Pathological lymph node metastases are frequently observed after surgery in patients, and lymph node metastasis correlates closely with prognosis after therapy [2, 3].

Lymph node stromal cells, particularly fibroblastic reticular cells, provide important structural support and regulate the immunity, tolerance and transport properties of lymph nodes [4]. While many studies have compared cancer-associated fibroblasts and nontumor fibroblasts, less is understood about the stromal contribution of metastatic lymph node fibroblasts (LN-Fbs) to the evolving microenvironment. A few reports thus far have provided mechanistic or signaling data related to LN-Fbs and immune cells [5]. However, whether LN-Fbs interact with tumor cells and protect tumor cells from chemotherapy is unclear. The purpose of this research was to characterize LN-Fbs and explore the interactions between LN-Fbs and tumor cells.

Chemotherapy resistance is responsible for tumor recurrence and represents one of the major challenges in oncology [6]. Here, we report a previously unrecognized function of metastatic lymph node fibroblasts during cancer progression and chemotherapy. We investigated the role of metastatic lymph node fibroblasts in ESCC and their implications in the response to cisplatin therapy. LN-Fbs secreted increased levels of high-molecular-weight PI16 (peptidase inhibitor 16) and promoted cisplatin resistance in ESCC tumor cells. These data demonstrate that LN-Fbs can transmit PI16 into ESCC cells and promote resistance in response to cisplatin. Identifying signaling events related to PI16 will provide possible new insights into therapeutic targeting of chemoresistance in ESCC tumor cells.

## Materials and methods

### Patients and tissue samples

This study was approved by the Institutional Review Board of Sun Yat-sen University Cancer Center (SYSUCC). For fibroblasts isolation, tumor tissues, nontumor tissues and metastatic lymph nodes were obtained from patients with ESCC who were not pretreated before surgery. For correlation analysis, 34 pairs of ESCC tumor tissues and metastatic lymph nodes were collected at SYSUCC. For chemoresistance analysis, 50 ESCC tumor tissues were collected from patients receiving platinum-based chemotherapy after surgery at SYSUCC. These samples are not overlapped with the above mentioned samples. None of the patients received neoadjuvant therapy. Patient characteristics are included in the supplemental data. Written informed consent was obtained from patients. The study was carried out in accordance with the Declaration of Helsinki guidelines.

### Isolation of fibroblasts and primary cell culture

Fibroblasts were isolated from primary tumors, noncancerous tissues, and matched metastatic mediastinal lymph nodes as described previously [7, 8]. Tissues were prewashed with 1× penicillin-streptomycin (NCM Biotech, China), minced into small pieces of approximately 1 mm^3^, and digested with Collagenase IV (Sigma-Aldrich, Germany). The digested suspension was centrifuged at 180 *g* and washed with DMEM twice. Then, the cell pellet was resuspended and cultured in DMEM supplemented with 20% fetal bovine serum, penicillin-streptomycin, levofloxacin (Beijing Pharm. Co. Ltd., China) and fluconazole (Pfizer Inc., NY). After incubating for 30 min at 37 °C, nonadherent cells were removed, and fibroblasts were obtained because of the different adhesion times of fibroblasts and tumor cells. This step was repeated several times until the tumor cells were completely removed. The fibroblasts were subcultured for further study.

### Cell lines

Esophageal squamous carcinoma cell lines, including KYSE510 (RRID: CVCL_1354), KYSE150 (RRID: CVCL_1348), KYSE410 (RRID: CVCL_1352), KYSE180 (RRID: CVCL_1349) and KYSE140 (RRID: CVCL_1347), were kindly provided by Dr. G Srivastava and Dr. GS Tsao of the University of Hong Kong [9]. 293FT cells (RRID: CVCL_6911) and NIH3T3 cells (RRID: CVCL_0594) were purchased from Invitrogen (Thermo Fisher Scientific, Waltham, MA). Mouse esophageal cancer cells (MEC2) were gift from Dr. L Fu of Senzhen University [10]. The cells were authenticated by STR profiling and confirmed to have no mycoplasma contamination.

All ESCC cells and fibroblasts were cultured in DMEM (Gibco BRL, NY) supplemented with 10% fetal bovine serum (Gibco BRL, NY) and incubated in a humidified atmosphere at 37 °C in 5% CO2.

### RNA sequencing and analyses

Total RNA was extracted from cells using TRIzol (Invitrogen, Carlsbad, CA). RNA yields were quantified using a Qubit™ RNA HS Assay Kit (Invitrogen, Carlsbad, CA), and RNA quality was assessed by 4200 TapeStation (Agilent, Santa Clara, CA). RNA sequencing was conducted by Haplox (Shenzhen, China). Sequences were generated with the Illumina novaseq 6000 platform, PE150 read format. For fibroblast analysis, differentially expressed gene (DEG) analysis (fold-change>1.2 or fold-change<0.83, *P* < 0.05) was implemented by DESeq2. For PI16 analysis, differential analysis was performed by EdgeR (fold-change>2 or fold-change<0.5, *P*adj<0.05). GO enrichment analysis was performed by using DAVID (https://david-d.ncifcrf.gov/) [11, 12]. KEGG pathway analysis was performed by using the Cluster Profiler R package [13].

### Conditioned medium (CM) and coculture experiments

For fibroblast conditioned medium, fibroblasts (1 × 10^6^) were grown in 10 cm dishes and cultured in 10 ml DMEM with 10% FBS for 24 h. The medium was collected and filtered through 0.45 µm filters [14].

For PI16 enriched medium, NIH3T3 cells stably transfected with pLVX-PI16 or vector were incubated with 10 ml serum-free DMEM for 24 h. The medium was collected, filtered through 0.45 µm filters, concentrated to 200 µl using a 10 kDa Amicon® Ultra-15 Centrifugal Filter Device (Millipore, Burlington, MA), flash-frozen in liquid nitrogen, and then stored at −80 °C for further experiments [14]. When used, the concentrate was added to fresh medium to 1 ml.

KYSE150 cells (5 × 10^3^ per well) were seeded in the bottom chamber of 24-well plates (Corning, NY), while NIH3T3 fibroblasts stably transfected with pLVX-PI16 or vector control (5 × 10^3^ per well) were seeded into the top chamber. A coculture system was established by inserting the top chamber into a 24-well plate.

### Liquid chromatography coupled with Orbitrap mass spectrometry (LC-MS/MS)

Fibroblasts at 80% confluence were pre-treated with 10 ml serum-free DMEM for 24 h. Supernatants were collected, filtered and concentrated with a 10 kDa Amicon® Ultra-15 Centrifugal Filter Device (Millipore, Burlington, MA). LC-MS/MS was conducted by Wininnovate Bio (Shenzhen, China). Protein digestion was carried out using the filter-aided sample preparation (FASP) method [15]. The peptide fractions were loaded into a nanoViper C18 (Acclaim PepMap 100, 75 μm × 2 cm) trap column. Online chromatography separation was performed on an Easy nLC 1200 system (Thermo Fisher, Waltham, MA) using a 90 min gradient on an analytical column (Acclaim PepMap RSLC, 75 μm × 25 cm C18-2 μm 100 Å). Data-dependent acquisition (DDA) mass spectrum techniques were used to acquire tandem MS data on a ThermoFisher Q Exactive mass spectrometer (Thermo Fisher, Waltham, MA) fitted with a Nano Flex ion source. The data were analyzed for protein identification and quantification using PEAKS Studio 8.5 (Bioinformatics Solutions Inc., Waterloo, Canada). The local false discovery rate at PSM was 1.0% after searching against the *Homo sapiens* database with a maximum of two missed cleavages. The following settings were selected: oxidation, acetylation, deamidation, pyro-glu from E, pyro-glu from Q for variable modifications and fixed carbamidomethylation of cysteine. Precursor and fragment mass tolerance were set to 10 ppm and 0.05 Da, respectively.

### Clinical analysis of public datasets and online bioinformatics tools

The Cancer Genome Atlas (TCGA) Esophageal Cancer (ESCA) transcriptome data were downloaded from the UCSC genomic center. The data from a total of 68 ESCC patients (<70 years of age at time of surgery) were used to analyze overall survival, and the patients were grouped based on a cutoff (log_2_^FPKM+1^: 12.27) for *PI16*. The Survminer R package was used to calculate the cutoff value for *PI16*.

GO enrichment analysis was performed by using DAVID (https://david-d.ncifcrf.gov) software [12]. Tumor purity and the correlation between *PI16* and ACTA2 in ESCA were calculated with Tumor Immune Estimation Resource (TIMER) (https://cistrome.shinyapps.io/timer/) [16]. Cell-type enrichment analysis was carried out on transcriptome data using the xCell cell type enrichment score tool (http://xcell.ucsf.edu/) [17] based on Tirosh signatures, including fibroblasts, endothelial cells, macrophages and B cells.

Single-cell RNA (scRNA) sequencing data (GSE134355 and PRJNA554845) were downloaded from GEO or Bioproject. The cell type composition of major human organs and the human cell landscape were constructed based on scRNA data. The Seurat R package was used to perform clustering analysis. 2D plots of single cells were visualized with the UMAP algorithm.

### Immunohistochemistry (IHC)

Paraffin-embedded formalin-fixed sections were deparaffinized with xylene, and rehydrated with a series of ethanol concentrations, then incubated with 3% hydrogen peroxide. Antigen retrieval was performed using Sodium Citrate Antigen Retrieval Solution (Boster, China) for 8 min. After blocked with 4% BSA, the sections were incubated with primary antibodies at 4 °C overnight, then with secondary antibody for 1 h at room temperature. The staining was visualized by DAB (Dako REAL™ EnVision™ Detection System, DAKO, Glostrup, Denmark). The degree of immunostaining was evaluated by two pathologists with no prior knowledge of patient characteristics. The staining intensity was scored as four levels: negative (0), weak (1), medium (2), and strong (3). The proportion of immunopositive cells in fibroblasts was scored as: 0–25% (1), 25–50% (2), 50–75% (3), 75–100% (4). The judgment of fibroblasts excluded muscle cells, vascular smooth muscle cells and cells of muscle bundle structure and tubular structure. The IHC score was obtained by multiplying the intensity score by the proportion score. PI16 high expression was defined as IHC score>3.

Additional methodology is provided in the Supplementary information.

## Results

### Isolation and RNA sequencing of fibroblasts derived from different tissues

**Fig. 1:**
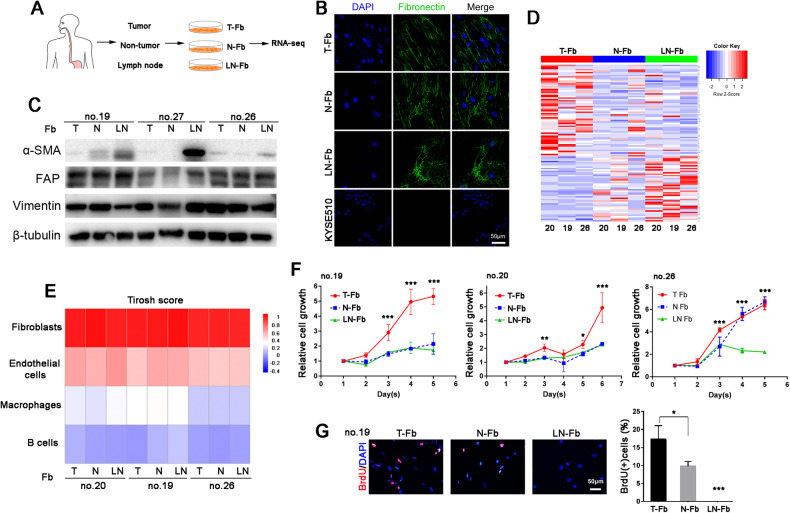
Characterization of fibroblasts derived from metastatic lymph nodes. **A** Schematic of the isolation of fibroblasts from tumor tissues (T-Fbs), nontumor tissues (N-Fbs) and metastatic lymph node tissues (LN-Fbs). **B** Representative images of fibronectin IF staining in fibroblasts and ESCC cells (KYSE510). **C** Western blotting results for α-SMA, FAP and vimentin in T-Fbs, N-Fbs and LN-Fbs in 3 ESCC patients. β-tubulin was used as a loading control. **D** Heatmap generated using hierarchical clustering of the transcriptome file of T-Fbs, N-Fbs and LN-Fbs in 3 ESCC patients. **E** Tirosh score generated by xCell and cell-type enrichment analysis results of transcriptome files of (**D**). **F** Relative cell growth curves of T-Fbs, N-Fbs and LN-Fbs in 3 ESCC patients (^*^P < 0.05; ^**^P < 0.01, ^***^P < 0.001; *P* value was calculated between T-Fbs and LN-Fbs). **G** Representative images and summary of BrdU incorporation results of T-Fbs, N-Fbs and LN-Fbs in patient no. 19 (^*^P < 0.05; ^***^P < 0.001).

Tumor fibroblasts (T-Fbs), matched nontumor fibroblasts (N-Fbs) and matched metastatic lymph node fibroblasts (LN-Fbs) were isolated from primary ESCCs by short-term primary culture in DMEM with 10% fetal bovine serum (Fig. 1A and Supplementary Fig. 1A). Positive staining of fibronectin was observed in fibroblasts in primary culture, whereas negative staining was observed in KYSE510 cells (Fig. 1B). We also tested some reported fibroblast markers, including α-SMA, FAP (fibroblast activation protein) and vimentin, in fibroblasts by western blotting. We detected increased expression of α-SMA in lymph node fibroblasts (Fig. 1C). Fibroblasts were also suspended and cultured in Matrigel [18] and similar results were observed (Supplementary Fig. 1B, C). We generated transcriptional profiles of T-Fbs, N-Fbs and LN-Fbs from 3 ESCC patients (no. 20, no. 19 and no. 26) and found 630 significantly upregulated genes in LN-Fbs compared to T-Fbs and 341 in LN-Fbs compared to N-Fbs (*P* < 0.01) (Fig. 1D and Supplementary Fig. 2). All transcriptomes were also assessed by utilizing a computational algorithm, xCell. The cell-type enrichment analysis of bulk transcriptomes defined the cells as fibroblasts (Fig. 1E). RNA-seq data of 10 cases of TCGA EC tissues were also analyzed and Tirosh score was generated in Supplementary Fig. 3.

Consistent with published reports [19, 20], gene signatures of activation of the MAPK pathway, inflammatory response and angiogenesis were significantly enriched in T-Fbs compared to N-Fbs (Supplementary Fig. 2B). Compared to T-Fbs, LN-Fbs exhibited upregulated expression of genes encoding molecules known to dampen cell proliferation, or to be involved in the response to toxic substances (Supplementary Fig. 2B). Cell chemotaxis and inflammatory response pathways were significantly enriched in LN-Fbs compared to N-Fbs (Supplementary Fig. 2B). We compared the cell growth of fibroblasts and found that LN-Fbs grew significantly slower than T-Fbs (Fig. 1F). The results were confirmed in the BrdU incorporation assay. The number of BrdU-positive cells was significantly decreased in LN-Fbs (Fig. 1G).

### Coculture with LN-Fbs conditioned medium inhibits ESCC tumor cell growth

**Fig. 2:**
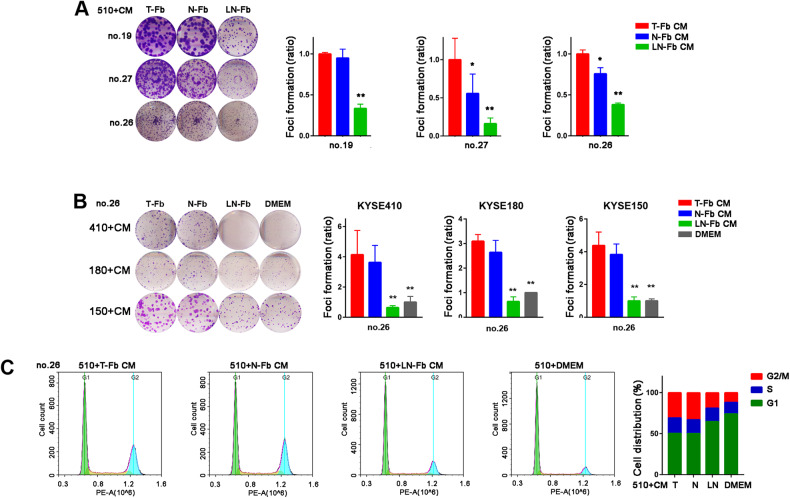
Coculturing with LN-Fbs inhibits ESCC tumor cell growth. **A** Representative images and summary of focus formation results of KYSE510 cells cocultured with T-Fbs, N-Fbs and LN-Fbs derived from different ESCC patients (^*^P < 0.05; ^**^P < 0.01). **B** Representative images and summary of KYSE410, KYSE180 and KYSE150 cells cocultured with T-Fbs, N-Fbs and LN-Fbs derived from an ESCC patient (no. 26) or a blank control (DMEM) (^**^P < 0.01). **C** Representative FACS plots and summary of the DNA content of KYSE510 cells cocultured with T-Fbs, N-Fbs and LN-Fbs from ESCC patient for 20 min (no. 26). (CM, conditioned medium).

We next assessed whether coculture with LN-Fbs affected the proliferation of ESCC cells. Cell-conditioned culture media of fibroblasts derived from different tissues were collected and added to ESCC cells. The focus formation assay results demonstrated that compared to coculture with T-Fbs conditioned medium (CM), coculture with LN-Fbs CM significantly inhibited KYSE510 cell proliferation (Fig. 2A). The results were confirmed in KYSE410, KYSE180 and KYSE150 cells cocultured with CM from LN-Fbs (Fig. 2B). Flow cytometric analysis results revealed a significant decrease in cell number in the G2/M phase in KYSE510 cells cocultured with LN-Fbs CM (Fig. 2C).

### Coculturing with LN-Fbs conditioned medium induces chemoresistance in ESCC cells

**Fig. 3:**
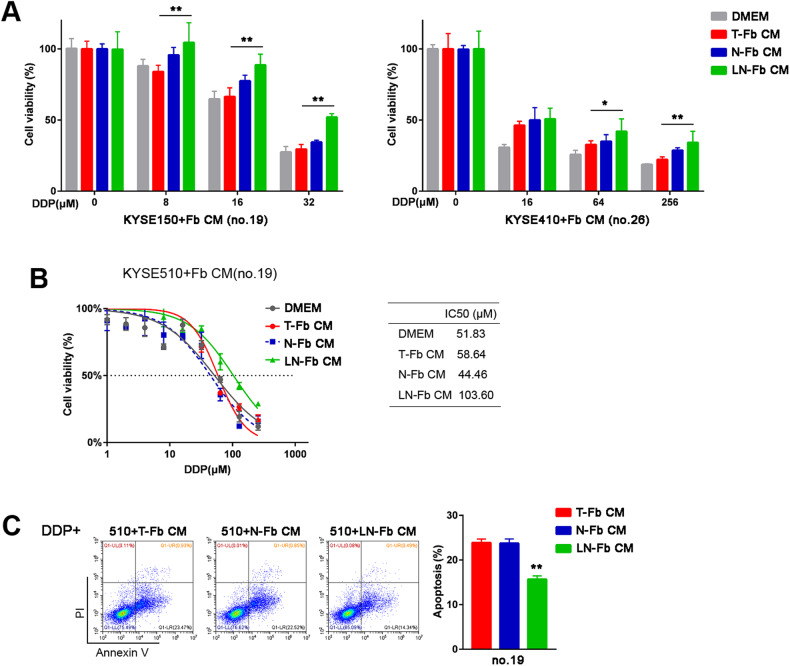
Coculturing with LN-Fbs induces resistance to cisplatin in ESCC cells. **A**, **B** Cell viability assay of KYSE150, KYSE410 and KYSE510 cells cocultured with T-Fbs, N-Fbs and LN-Fbs from ESCC patients. Then, the cells were treated with different concentrations of DDP (^*^P < 0.05; ^**^P < 0.01). **B** The half maximal inhibitory concentration is listed (*right*). **C** Representative images and summary showing the apoptotic cell percentage of KYSE510 cells cocultured with T-Fbs, N-Fbs and LN-Fbs derived from ESCC patients and treated with 20 μM DDP for 12 h.

Because the response to toxic substances pathway was enriched in LN-Fbs, we next investigated whether drug resistance is induced in ESCC cells cocultured with LN-Fbs CM. The cell viability results demonstrated that ESCC cells cocultured with LN-Fbs CM were more resistant to cisplatin (DDP) than cells cocultured with T-Fbs CM (Fig. 3A and Supplementary Fig. 4). The half maximal inhibitory concentration (IC50) of DDP was assayed in KYSE510 cells cocultured with different conditioned media of fibroblasts. The IC50 value was significantly increased in KYSE510 cells cocultured with LN-Fbs CM compared with DMEM or other Fbs CM (Fig. 3B). Flow cytometric analyses revealed a significant decrease in the apoptosis rate in ESCC cells cocultured with LN-Fbs CM when cells were treated with DDP (Fig. 3C). Other chemotherapeutic agents including 5-Fu and Paclitaxel were also tested and no significant difference was observed between ESCC cells cocultured with LN-Fbs and ESCC cells cocultured with T-Fbs (Supplementary Fig. 5).

### LN-Fbs induce chemoresistance to DDP in ESCC cells via PI16

**Fig. 4:**
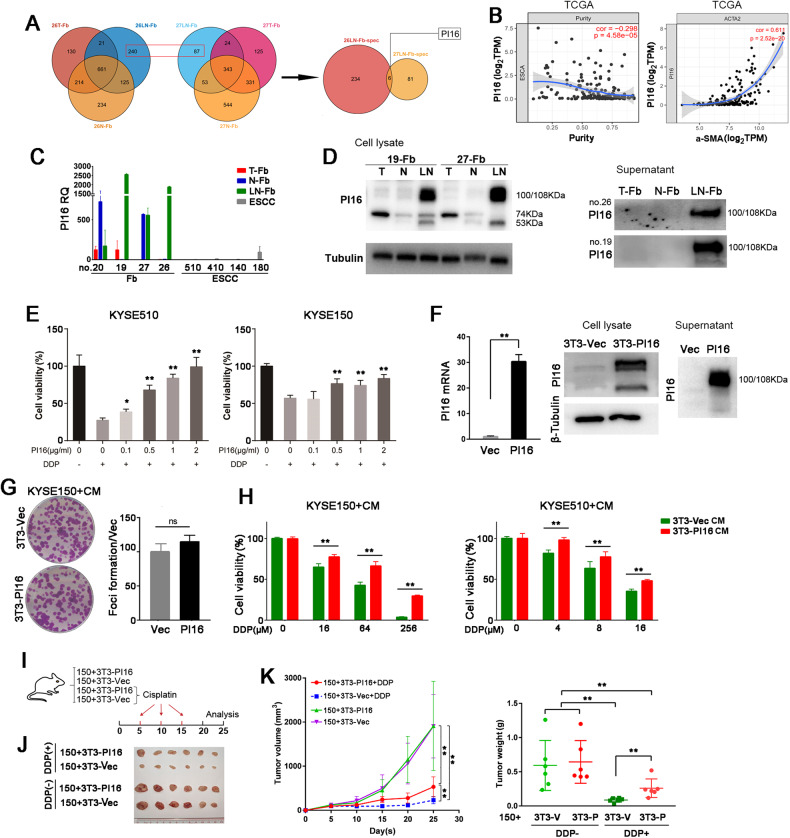
LN-Fbs induce resistance to DDP via PI16. **A** Flow chart of PI16 screened in LN-Fbs derived from different ESCC cases. **B** Correlation analyses between PI16 and tissue purity (*left*) or ACTA2 (α-SMA) (*right*) using TIMER. **C** qRT-PCR results of PI16 in ESCC cell lines and fibroblasts derived from different tissues. **D** Western blotting results of PI16 in the cell lysate and concentrated culture medium of T-Fbs, N-Fbs and LN-Fbs derived from an ESCC patient (no. 26). **E** Cell viability of ESCC cells treated with PI16 and DDP. **F** The mRNA and protein levels of PI16 were measured by qRT-PCR (*left*) and western blotting in 3T3-PI16 and control cells (*middle*: cell pellet; *right*: supernatant). **G** Focus formation results of KYSE150 cells cocultured with 3T3-PI16 and control cells. **H** Cell viability of ESCC cells cocultured with 3T3-PI16 or control cells. Cells were then treated with different concentrations of DDP. **I** Graphical scheme describing the workflow of animal experiments. Image (**J**), tumor growth curve and tumor weight of xenografts (**K**) formed in nude mice. (ns: not significant; ^**^P < 0.01).

To investigate the mechanism by which coculturing with LN-Fbs induces drug resistance in ESCC cells, we collected the conditioned medium of two sets of fibroblasts (including T-Fbs, N-Fbs and LN-Fbs) from patients 26 and 27. LC-MS/MS was performed, and specific peptides were identified in 26LN-Fbs CM (240 peptides) and 27LN-Fbs CM (87 peptides). The peptides of the two LN-Fbs were intersected, and PI16 was screened out in the assay (Fig. 4A). We explored the TCGA database (esophageal carcinoma) using an online tool: Tumor Immune Estimation Resource (TIMER) (https://cistrome.shinyapps.io/timer/). The analyses revealed that PI16 was negatively correlated with ESCC tumor tissue purity (correlation = −0.298, *P* < 0.001) and positively correlated with α-SMA (correlation = 0.611, *P* < 0.001) (Spearman’s correlation) (Fig. 4B). Consistent with the finding that PI16 is mainly expressed in fibroblasts (the data were extracted from GEO (GSE134355) or Bioproject (PRJNA554845), and the map was drawn with UCSC Cell Browser (Supplementary Fig. 6), we conducted qPCR on fibroblasts and ESCC cells and found that PI16 was highly expressed in LN-Fbs and remained at a low level in ESCC cells. In addition, compared with LN-Fbs, PI16 was at a relatively lower level in T-Fbs (Fig. 4C). We noticed that PI16 in the cell lysate had three electrophoretic bands (100/108 kDa, 74 kDa and 53 kDa) according to western blotting, which was consistent with a previous report [21]. LN-Fbs exhibited significantly increased the largest isoforms of PI16 (100/108 kDa) (Fig. 4D). The cell culture medium was collected and western blotting results demonstrated that PI16 (100/108 kDa) is highly expressed in fibroblasts derived from metastatic lymph nodes (Fig. 4D). To validate the effect of PI16 on drug resistance, we added PI16 recombinant protein into the culture medium of ESCC cells. We found that PI16 addition significantly increased drug resistance in ESCC cells (Fig. 4E). We then established a stable NIH3T3 cell line with PI16 overexpression (3T3-PI16). The mRNA and protein levels were validated in 3T3-PI16 and vector control cells (Fig. 4F). The cell culture medium was also collected and detected by western blotting (Fig. 4F). The focus formation assay results demonstrated that coculturing with 3T3-PI16 conditioned medium had no significant impact on the proliferation of ESCC cells (Fig. 4G). Consistent with the PI16 recombinant protein effect, ESCC cells cocultured with 3T3-PI16 CM demonstrated significantly increased resistance to DDP (Fig. 4H). An in vivo xenograft assay was carried out by subcutaneously inoculating KYSE150 cells mixed with 3T3-PI16 or 3T3-Vec cells into nude BALB/c mice. The mice then received cisplatin treatment or not (Fig. 4I). Coinjecting with 3T3-PI16 did not impact ESCC xenografts growth or tumor weight, however, it increased the drug resistance of ESCC cells, as the tumor volume and tumor weight were significantly higher in KYSE150 cells mixed with 3T3-PI16 than in KYSE150 cells mixed with 3T3-Vec when both groups were treated with cisplatin (Fig. 4J, K). These results suggested that fibroblasts from lymph nodes may confer DDP resistance to ESCC cells by secreting PI16. We also tested whether PI16 could increase resistance to radiation by exposing ESCC cells to different doses of radiation. No significant difference was observed between cells cocultured with CM from 3T3-PI16 cells and cells cocultured with CM from control cells (Supplementary Fig. 7).

### Coculturing with 3T3-PI16 regulates p38 and JNK in response to cisplatin treatment

**Fig. 5:**
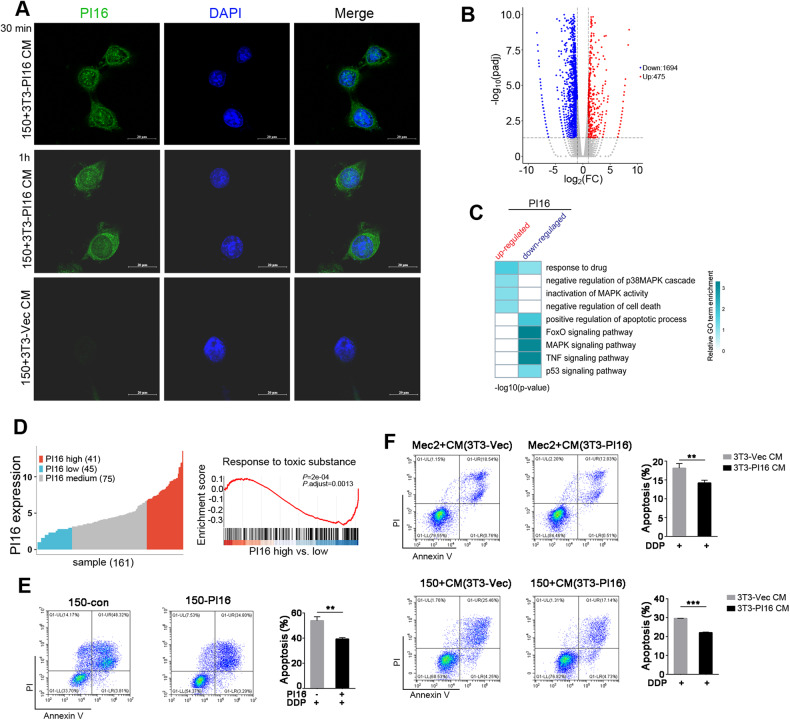
Intracellular uptake of PI16 by ESCC cells decreases cisplatin-induced apoptosis. **A** Immunofluorescence staining of PI16 in KYSE150 cells cocultured with CM from 3T3-PI16 cells for 30 min (*top*) and 1 h (*middle*) or CM from 3T3-Vec cells for 1 h (*bottom*). (PI16, green; DAPI: blue). **B** Volcano plot of genes upregulated (*red*) or downregulated (*blue*) in KYSE150 cells cocultured for 1 h with recombinant protein PI16 compared to the vehicle control. **C** Heatmap displaying enrichment of Gene Ontology (GO) terms in PI16 upregulated genes and downregulated genes from Fig. 5B. **D** Quartile and GSEA results of PI16 high expression vs. low expression in the TCGA ESCA database. **E** Representative images and summary of apoptosis in KYSE150 cells treated with recombinant protein PI16 or vehicle control and cisplatin. **F** Representative images of apoptosis in MEC2 or KYSE150 cells cocultured with CM from 3T3-PI16 or 3T3-Vec cells and cisplatin (^**^P < 0.01; ^***^P < 0.001).

To investigate whether ESCC cells could take up PI16 when coculturing with fibroblasts, KYSE150 cells were cocultured with CM from 3T3-PI16 or vector control cells. Immunofluorescence staining revealed positive staining of PI16 in ESCC cells cocultured with CM from 3T3-PI16 at 30 min or 1 h (Fig. 5A). We also set up a direct coculturing system by plating NIH3T3 derivative cells in the top chamber and ESCC cells in the bottom chamber (Supplementary Fig. 8A). Similar results were observed in direct cocultures of KYSE150 cells and 3T3-PI16 cells (Supplementary Fig. 8B). We next treated KYSE150 cells with recombinant human PI16 or vehicle for 1 h, and RNA-seq was performed on PI16-treated cells and control cells. Analysis of transcriptional profiles revealed that 1694 genes were downregulated and 475 genes were upregulated in the PI16-treated group (Fig. 5B). We further characterized the gene expression induced by PI16. Gene Ontology term enrichment analysis within PI16 upregulated genes or downregulated genes revealed enrichment of pathways of apoptosis, MAPK signaling pathway and response to drugs (Fig. 5C). In 161 ESCC tumor tissues from the TCGA ESCA database, the tissues were distributed according to quartiles of *PI16* mRNA levels (Fig. 5D). GSEA of the top quartile compared to the bottom quartile revealed that response to toxic substances was enriched (Fig. 5D), which is consistent with Fig. 5C.

Next, KYSE150 cells were treated with recombinant protein PI16 and cisplatin. The apoptosis rate was significantly decreased in PI16-treated ESCC cells (Fig. 5E). Consistent with this, the number of apoptotic cells was decreased in mouse esophageal cancer cells (MEC2) and KYSE150 cells cocultured with CM from 3T3-PI16 cells compared to 3T3-Vec cells (Fig. 5F).

**Fig. 6:**
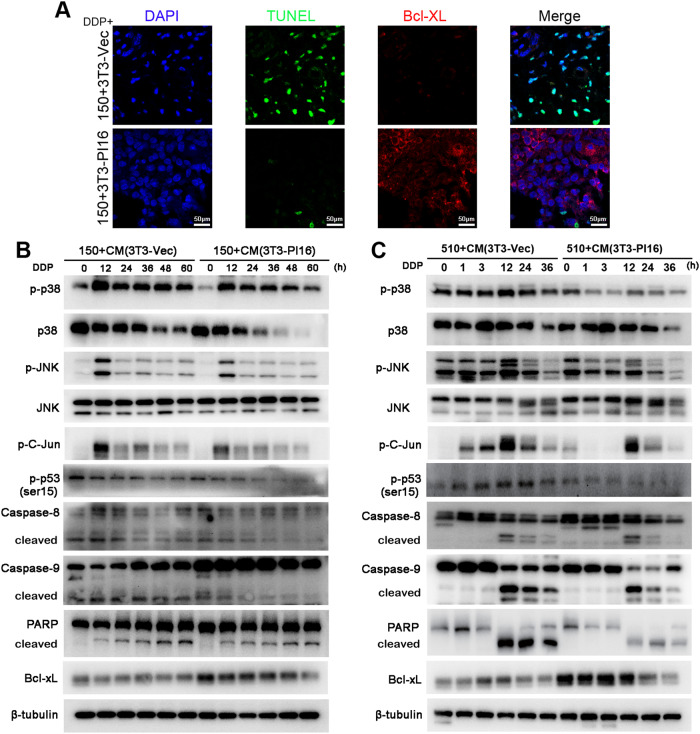
Coculturing with 3T3-PI16 regulates p38 and JNK in response to DDP. **A** Immunofluorescence staining of TUNEL and Bcl-XL in xenograft sections of KYSE150 cells mixed with 3T3-PI16 or 3T3-Vec cells treated with cisplatin (animal experiments in Fig. 4J) (TUNEL, green; Bcl-XL, red; DAPI, blue). Western blotting results of proteins in KYSE150 (**B**) and KYSE510 (**C**) cells cultured in CM of 3T3-PI16 or 3T3-Vec cells in the presence of cisplatin for different times.

Immunofluorescence analyses of TUNEL and Bcl-xL were performed on xenografts of KYSE150+3T3-PI16 and KYSE150+3T3-Vec animals treated with cisplatin (Fig. 4I). The number of TUNEL-positive cells was decreased and that of Bcl-xL-positive cells was increased in KYSE150+3T3-PI16 xenografts compared with KYSE150+3T3-Vec xenografts (Fig. 6A). To further explore the mechanism of drug resistance of ESCC cells conferred by 3T3-PI16 against cisplatin, western blotting analyses were performed on KYSE150 and KYSE510 cells cocultured with CM from 3T3-PI16 and 3T3-Vec in the presence of cisplatin. The results revealed that phosphorylation of p38, JNK, C-Jun and P53 (ser15) was decreased significantly in ESCC cells cocultured with CM from 3T3-PI16 cells compared to CM from 3T3-Vec cells in response to cisplatin (Fig. 6B, C). These results indicated that coculturing with CM from 3T3-PI16 cells regulated the ESCC cell response to cisplatin through p38 and JNK inhibition. The expression of cleaved caspase 8, caspase 9 and PARP was found to be reduced in ESCC cells cocultured with 3T3-PI16 CM compared to 3T3-Vec CM (Fig. 6B, C). We also examined the expression of Bcl-XL, and significantly increased expression of Bcl-XL was detected in ESCC cells cocultured with 3T3-PI16 CM compared to that with3T3-Vec cells upon cisplatin treatment (Fig. 6B, C). In addition, γH2AX was detected in ESCC cells cocultured with CM from 3T3-PI16 or 3T3-Vec cells and cells were treated with cisplatin. No significant difference was observed between the experimental group and the control group (Supplementary Fig. 9).

### Clinical significance of PI16 in ESCC

**Fig. 7:**
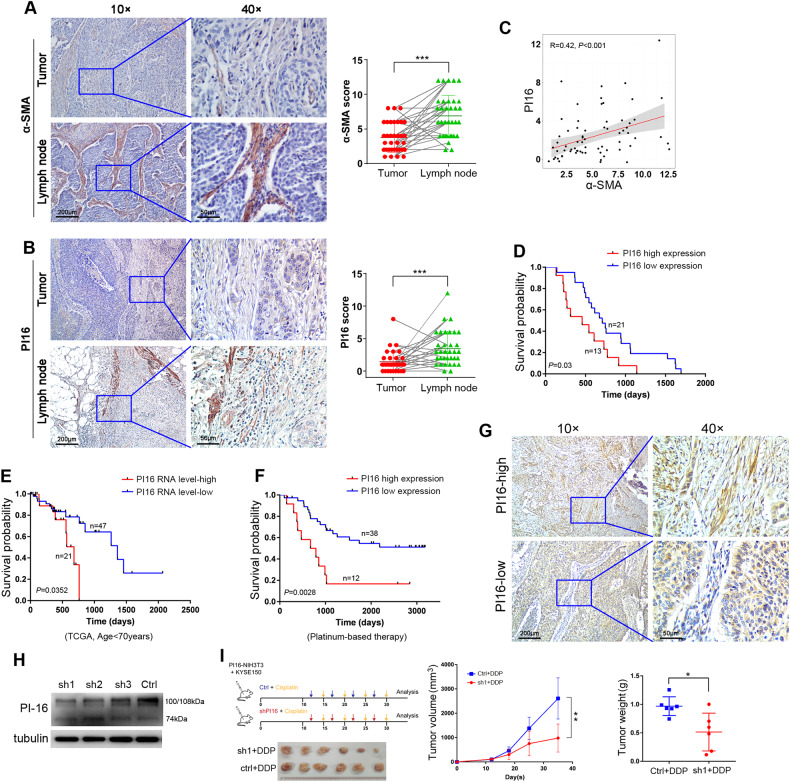
Clinical significance of PI16 in ESCC and potential use of shRNA-PI16 in vivo. Representative images and IHC score summaries of α-SMA (**A**) and PI16 (**B**) staining of ESCC tumor tissues and metastatic lymph nodes. The square on the left panel is magnified in the right panel (^***^P < 0.001). **C** The correlation of α-SMA with PI16 in tumor and metastatic lymph nodes. **D** The survival curves of the PI16 high and low expression groups in fibroblasts of metastatic lymph nodes of ESCC patients (*P* = 0.03, log-rank). **E** Kaplan–Meier plot of overall survival in ESCC patients stratified by *PI16* mRNA level in tumor tissues (TCGA dataset, age<70 years at time of surgery) (*P* = 0.0352, log-rank). **F** Kaplan–Meier plot of overall survival in ESCC patients stratified by PI16 protein expression in fibroblasts of tumor tissues (*P* = 0.0028, log-rank). Patients underwent platinum-based chemotherapy after surgery. **G** Representative IHC images of PI16 in interstitial tissues in ESCC tumors. The boxed regions are magnified as images on the right. **H** Western blotting results of PI16 in 3T3-PI16 cells transfected with shRNAs targeting PI16 or scramble RNA (control). **I** Graphical scheme describing the workflow of animal experiments (*left*). Tumor growth curve (*middle*) and tumor weight (*right*) of the xenografts of the mice receiving the combination of DDP with shRNA targeting PI16 or control.

We used IHC staining to examine the α-SMA protein expression level in 34 pairs of ESCC tumor tissues and metastatic lymph nodes collected at Sun Yat-sen University Cancer Center (SYSUCC). α-SMA protein expression significantly increased in fibroblasts of lymph nodes compared to paired tumor tissues (*P* < 0.001) (Fig. 7A). We also found that PI16 was upregulated in fibroblasts of metastatic lymph nodes compared to tumor tissues (*P* < 0.001) (Fig. 7B). We further tested the correlation between α-SMA and PI16 expression in fibroblasts of these ESCC tumor tissues and metastatic lymph nodes and observed a positive correlation between α-SMA and PI16 expression (R = 0.42, *P* < 0.001, Spearman’s) (Fig. 7C). Survival analysis was also performed in these patients according to PI16 expression in fibroblasts of lymph nodes. Patients with high PI16 expression in Fbs of lymph nodes showed poorer survival than patients with low PI16 expression (*P* = 0.03; log-rank) (Fig. 7D). The multivariate analysis showed that PI16 upregulation in Fbs of lymph nodes was a marginal independent prognostic factor for worse survival in ESCC patients (Supplementary table 1). The median survival was 709 days in the low PI16 expression group and 457 days in the high PI16 expression group.

Because PI16 is mainly expressed in fibroblasts (Fig. 4C and Supplementary Figure 6), we next investigated the prognostic value of PI16 RNA level in the TCGA ESCC database. High *PI16* mRNA levels were correlated with shorter overall survival in 68 patients with ESCC (patients were under 70 years old at the time of surgery) in the TCGA ESCC database (*P* = 0.0352, log-rank, Fig. 7E). The median survival was 1361 days in the low *PI16* level group and 681 days in the high *PI16* level group. PI16 upregulation at the mRNA level also independently predicted worse survival of ESCC patients (Supplementary table 2). Because the above results showed that PI16 overexpression in fibroblasts induced resistance to cisplatin, we further collected ESCC samples from 50 ESCC patients receiving platinum-based therapy postsurgery at SYSUCC and examined PI16 expression in interstitial tumor samples using IHC staining. ESCC patients with high PI16 expression in tumor interstitial tissues exhibited worse survival than patients with low PI16 expression (*P* = 0.0028, log-rank) (Fig. 7F, G). The survival analysis demonstrated that the 3-year survival rates were 66.8% in the low PI16 expression group and 16.7% in the high PI16 expression group. Multivariate analysis indicated that high PI16 expression in tumor interstitial tissues was an independent prognostic factor for ESCC patients undergoing platinum-based chemotherapy (Supplementary table 3).

### Knockdown of PI16 in Fbs sensitizes ESCC cells to cisplatin in vivo

To substantiate the findings in PI16-mediated cisplatin resistance, shRNAs targeting PI16 were screened by western blotting (Fig. 7H). We first established xenografts by subcutaneously inoculating KYSE150 cells with 3T3-PI16 cells into nude mice. The mice were then treated with cisplatin and intratumoral injection of shRNA-1 targeting PI16 or scramble control (Fig. 7I). Tumor growth curves and tumor weights showed that shRNA-1 targeting PI16 in fibroblasts significantly increased the sensitivity of ESCC cells to cisplatin and significantly decreased the xenograft growth (Fig. 7I). No significant difference was observed in the amount of fibroblasts between the shRNA-1 treated group and the control group (Supplementary Fig. 10).

We next explored whether p38 activation could increase sensitivity to cisplatin. Xenografts were established as described above. Animals were intratumorally injected with Dehydrocorydaline chloride (DHC), a p38 activator, or vehicle control. All animals were treated with cisplatin. The results demonstrated that 3T3-PI16 significantly increased cisplatin resistance of ESCC cells and p38 activation reversed the effect and enhanced cisplatin sensitivity in ESCC cells (Supplementary Fig. 11).

**Fig. 8:**
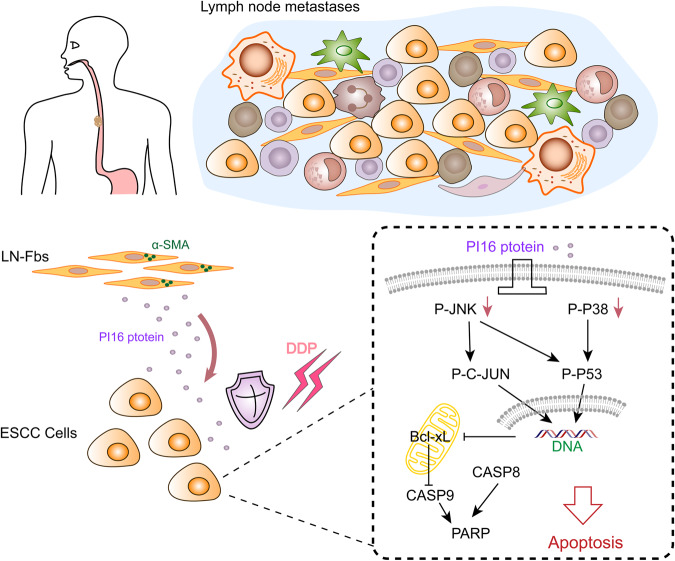
Mechanisms by which LN-Fbs secrete PI16 and confer chemoresistance to ESCC cells. Fibroblasts in metastatic lymph nodes secrete PI16, regulate p38 and JNK pathways, decrease cell apoptosis and confer cisplatin chemoresistance to ESCC cells.

In conclusion, our study reveals a functionally important mechanism of fibroblasts in metastatic lymph nodes of ESCC. Our results suggest a model in which fibroblasts in metastatic lymph nodes decrease apoptosis of ESCC cells via PI16 when cells are treated with cisplatin, thereby providing a cisplatin-resistance niche and supporting ESCC tumor cell survival in metastatic lymph nodes (Fig. 8).

## Discussion

Cancer-associated fibroblasts (CAFs) have been implicated as protumorigenic in many cancers [7, 18]. Fibroblasts in lymph nodes are reported to inhibit the proliferation of activated T cells in a contact-dependent manner [22–24]. Our research demonstrates that fibroblasts isolated from metastatic lymph nodes subdue the proliferation of cocultured ESCC cells. In addition, coculturing with LN-Fbs confers cisplatin resistance to ESCC cells. By integrating RNA-sequencing data with secretome profiling data by mass spectrometry from LN-Fbs vs. T-Fbs vs. N-Fbs, PI16 was identified in fibroblasts derived from metastatic lymph nodes, which is also one of the dominant cluster-specific annotation genes according to single-cell transcriptomic data from fibroblasts across different tissues, datasets and disease states [25].

PI16 received its name from a sequence homology to peptidase inhibitor 15, which has been found to inhibit trypsin [26]. The peptide sequence classifies PI16 as a member of the CAP (cysteine-rich secretory proteins, antigen 5, and pathogenesis-related 1 proteins) superfamily, which exhibits diverse functions and is not well studied [27]. It has been reported that PI16 appears in at least three electrophoretic isoforms, each corresponding to an apparent mass that is higher than its theoretical mass (100 kDa and 108 kDa, 74 kDa and 53 kDa) [21]. In our research, we also found 2–3 bands in the cell lysates of fibroblasts. However, a dramatic upregulation of the largest protein size (100/108 kDa) was found in fibroblasts derived from metastatic lymph nodes. It is the major protein size in the supernatant of LN fibroblasts, which was confirmed in human-derived LN-Fbs and PI16-overexpressing NIH3T3 cells. Taken together, these results suggest that 100/108 kDa PI16 is the major secretory protein of PI16 in fibroblasts.

A recent study indicated that PI16 could attenuate the response to sorafenib in hepatocellular carcinoma [28]. Our research demonstrates that PI16 secreted by LN-Fbs does not promote ESCC tumor cell proliferation, it elevates the refractoriness of tumor cells to cisplatin. Notably, high expression of PI16 exists in fibroblasts of metastatic lymph nodes compared with tumor-associated fibroblasts. Big data analysis and clinical samples collected at SYSUCC reveal that high expression of PI16 in bulk RNA or protein levels in fibroblasts predicts poor survival of patients with ESCC. We also found that PI16 secreted by fibroblasts could not protect ESCC cells from radiation and other chemotherapeutic reagents such as 5-Fu and paclitaxel in vitro.

The MAPK pathway has been shown to be important in mediating responses to cisplatin chemotherapy in various cancers [29]. Chemotherapeutic drugs, such as cisplatin, induce the activation of JNK and p38 in many cancers, and signaling pathway activation is critical in determining the cellular response to the drug [30]. Both JNK and p38 signaling pathways are either proapoptosis or prosurvival, depending on the cell type, the nature of the stimulus, the duration of the activation, and the interaction with other factors [29, 31]. In this study, p38 and JNK activation was impeded in response to cisplatin in ESCC cells cocultured with PI16-overexpressing fibroblasts. When p38 was activated, the resistance to cisplatin was reversed and sensitivity to cisplatin was increased in vivo study. In addition, reduced phosphorylation of the downstream proapoptotic transcription factor C-Jun and phosphorylation of p53 at Ser15 leading to decreased p53 accumulation were also involved in the resistance to cisplatin in ESCC cells cocultured with PI16-overexpressing fibroblasts. Although no difference of γH2AX was observed, the possibility of other mechanisms cannot be excluded.

To explore the therapeutic potential of PI16 as a target, we knocked down PI16 expression in combination with cisplatin in an in vivo assay and found that knockdown of PI16 increased the therapeutic effect of cisplatin in mice with xenografts formed by ESCC cells and fibroblasts. Our studies suggest the potential of novel therapeutic combinations to selectively modulate the microenvironment by targeting PI16-secreting fibroblasts, along with chemotherapy, such as cisplatin.

In this research, we isolated and established different fibroblasts from ESCC tumor tissues, nontumor tissues and metastatic lymph nodes. The fibroblasts of metastatic lymph nodes were also characterized. Furthermore, through RNA-seq and secretome assessment by mass spectrometry, we identified the LN-Fb-secreted molecule, namely, PI16 (100/108 kDa, high molecular weight), which is potentially responsible for providing chemoresistance to cisplatin in ESCC tumor cells in metastatic lymph nodes. The ability of LN-Fbs to regulate the responses of ESCC tumor cells to DDP constitutes an attractive area of investigation regarding a combined treatment that targets the tumor microenvironment and provides chemotherapy. In addition, knockdown of PI16 increased the efficacy of cisplatin in an in vivo assay. Moreover, PI16 is a novel biomarker for predicting poor clinical response to cisplatin treatment in patients with ESCC.

Overall, our findings provide novel insights into lymph node stroma - ESCC tumor cell communication and a potential therapeutic target to effectively block the LN-Fbs-enhanced tumor cell chemoresistance niche signaling circuit in response to cisplatin.

### Supplementary information

Supplementary information

## Data Availability

The materials described in the manuscript, including all relevant raw data, will be freely available to any researcher wishing to use them for non-commercial purposes, without breaching participant confidentiality.
