# Supplementary material for: Fibroblasts in metastatic lymph nodes confer cisplatin resistance to ESCC tumor cells via PI16
Source: Oncogenesis. 2023 Nov 1;12(1):50. doi: 10.1038/s41389-023-00495-x (PMC10620422; doi:10.1038/s41389-023-00495-x)
Supplement: Supplementary file 1 — Supplementary information [file 41389_2023_495_MOESM1_ESM.docx]

**Supplementary information:**

1 Supplementary materials and methods

2 Supplementary tables

3 Supplementary figures and figure legends

**Supplementary materials and methods:**

**Primers and shRNAs:**

| **Names** | **Sequences** |
| --- | --- |
| 18S rRNA | Forward: 5’-GTAACCCGTTGAACCCCATT-3’  Reverse: 5’-CCATCCAATCGGTAGTAGCG-3’ |
| PI16 | Forward: 5’- CTGGTGTGCAACTATGAGCCTC-3’  Reverse: 5’- GGCAAATCCTGAGCATCTTCCG-3’ |
| shRNA-mPI16-1 | 5’-CCTATCGCTCAAACAAGATAC-3’ |
| shRNA-mPI16-2 | 5’-CCAAAGAAATCGCTGTACCCC-3’ |
| shRNA-mPI16-3 | 5’-CCCAAATCTATGGACGAAGAA-3’ |

**Antibodies:**

| **Antibody** | **Vendor** | **Catalog No.** | **Application** |
| --- | --- | --- | --- |
| Fibronectin | BD Transduction  Laboratories | 610078 | IF |
| α-SMA | Cell Signaling Technology | 14968 | WB, IHC |
| FAP | Cell Signaling Technology | 66562 | WB |
| Vimentin | Santa Cruz Biotechnology | Sc32322 | WB |
| PI16 | Boster | A09887-1 | WB, IHC |
| p38 MAPK | Cell Signaling Technology | 9212 | WB |
| JNK2 | Cell Signaling Technology | 9258 | WB |
| Phospho-p38 MAPK | Cell Signaling Technology | 4511 | WB |
| Phospho-SAPK/JNK | Cell Signaling Technology | 9251 | WB |
| Phospho-c-Jun | Cell Signaling Technology | 3270 | WB |
| Phospho-p53 (Ser15) | Cell Signaling Technology | 9286 | WB |
| Bcl-xL | Cell Signaling Technology | 2764 | WB, IF |
| PARP | Cell Signaling Technology | 9542 | WB |
| Caspase-8 | Cell Signaling Technology | 9746 | WB |
| Caspase-9 | Cell Signaling Technology | 9502 | WB |
| β-Tubulin | Cell Signaling Technology | 86298 | WB |
| GAPDH | ABGENT | AM1020B | WB |
| Phospho-Histone H2A.X (Ser139) (20E3) Rabbit mAb | Cell Signaling Technology | #9718 | WB |
| Anti-mouse IgG（HRP） | Cell Signaling Technology | 7076 | WB |
| Anti- rabbit IgG（HRP） | Cell Signaling Technology | 7074 | WB |
| Anti-mouse IgG Alexa Fluor 488 | ThermoFisher | A11001 | IF |
| Anti- rabbit IgG Alexa Fluor 594 | ThermoFisher | A21203 | IF |

**Reagents:**

| **Reagents** | **Vendor** |
| --- | --- |
| Cisplatin | JiangSu Hansoh Pharmaceutical Company |
| 5-Fu | HaiNan Zhuotai Pharmaceutical Company |
| Paclitaxel | SiChuan Huiyu Pharmaceutical Company |
| Matrigel® Basement Membrane Matrix | Corning |
| Dehydrocorydaline chloride (DHC) | MedChemExpress |

**Plasmids and shRNAs**:

pLVX-PI16-3FLAG and control plasmids were purchased from GeneCopoeia (Guangzhou, China). Lentivirus-containing PI16 was packaged using the ViraPowerTM Lentiviral Packaging Mix (Invitrogen, Carlsbad, CA) in 293FT cells. NIH3T3 cells were transduced with lentivirus and stable cells were established by 2 μg/ml puromycin (Sigma-Aldrich) selection. Lentivirus psi-LVRU6GP containing shRNAs targeting mouse PI16 and control vectors were purchased from GeneCopoeia (Guangzhou, China). The titer of virus solution was 2×10^8^ TU/ml.

**Fibroblasts cultured in Matrigel**

Fibroblasts suspension (6×10^6^/ml in 30 μl medium) mixed with 270 μl Matrigel (5 mg/ml) were plated into 24-well plate that was coated with Matrigel (5 mg/ml). Thirty min later, 500 μl complete medium was added and cells were cultured for 7 days.

**Cell cycle analysis**:

Fibroblasts (5×10^5^) were cultured with serum-free DMEM for 24 h to synchronise the cell growth. Subsequently, cells were incubated with complete DMEM for 20 min, then digested with EDTA-free trpsin (Gibco BRL, Grand Island, NY). Cells were collected and fixed with 75% cold ethanol overnight at 4°C. The cells were washed by PBS and incubated with RNase at 37 °C for 30 min. After staining with PI solution, the cells were then analyzed by flow cytometry (Beckman Coulter, Fullerton, CA). Cell cycle analysis was performed by ModFir LT 3.1 (Beckman Coulter, Fullerton, CA).

**Apoptosis assay**:

Cells were treated with cisplatin (Hansoh Pharmaceutical Company, China) (20 µM for KYSE510 and MEC2, 40 µM for KYSE150) for indicated time. The cells were harvested and stained with Annexin-V Apoptosis Detection Kit (Dojindo, Japan). Flow cytometric analysis was performed with CytoFLEX (Beckman Coulter, Fullerton, CA). Three independent assays were performed.

**Immunofluorescence, TUNEL and BrdU assays**:

Cells were fixed, rinsed by PBS, and permeabilized with 0.1% Triton X-100 (Sigma-Aldrich, Germany) for 5 min. Cells were then blocked with 5% BSA followed by incubation with primary antibodies overnight at 4°C and with secondary antibody for 1 h at room temperature. The cells were washed and mounted with mounting medium containing DAPI (Abcam, Cambridge, UK).

TUNEL staining was performed using the In Situ Cell Death Detection Kit (Roche, Germany) following the manufacturer’s protocol. BrdU (5-bromo-2´-deoxy-uridine) incorporation assay was performed using BrdU Labeling and Detection Kit Ⅱ (Roche, Germany) following the manufacturer's instructions. Briefly, fibroblasts were incubated with BrdU labeling medium at 37°C, 5% CO_2_ for about 10 h. Cells were fixed and incubated with Anti-BrdU for 30 min at 37°C, followed by anti-mouse IgG incubation for 1 hour at room temperature. The cells were counterstained with DAPI (Abcam, Cambridge, UK). BrdU incorporation was measured under a fluorescent microscope.

**Western blotting**:

Western blotting analysis was performed according to the standard protocol. Protein lysates were resolved with SDS-polyacrylamide gel and transferred to a polyvinylidene fluoride membrane. Membranes were blocked with 5% BSA, incubated with primary antibodies at 4 °C overnight, followed by incubation with secondary antibody for 1 h at room temperature. Chemiluminescence signals were detected by the ChemiDoc Touch Imaging System (Bio-Rad, Hercules, CA).

**Quantitative polymerase chain reaction (qPCR)**:

Total RNA was extracted using TRIzol Reagent (Invitrogen, Carlsbad, CA) or a rapid RNA extraction kit (ES Science, China) according to the manufacturer's protocol, and reverse transcription was carried out using PrimeScript RT Master Mix Kit (TakaRa, Shiga, Japan). The qPCR was performed using FastStart Universal SYBR Green Master (Roche, Germany) and run on a Roche 480 Real-Time PCR system (Roche, Germany). 18s was used as a control for normalization of the expression levels. The relative quantification was calculated by 2^−ΔΔCt^.

**Cell growth assay:**

Cell growth assays were performed using Cell Counting Kit-8 (Dojindo, Kumamoto, Japan) according to the manufacturer's manual. Cells were plated into 96-well plates at 1×10^3^ per well and cultured for 7 days. The cells were incubated with CCK-8 and the absorbance at 450 nm was measured every day. The experiments were repeated three times independently.

**Focus formation assay:**

Cells were seeded at low density into 6-well plates and cultured for 10-14 days. Cell colonies (>50 cells/colony) were fixed, stained with 1% crystal violet and counted. The experiments were repeated three times independently.

**Animal experiments**:

All animal experiments were approved by the Animal Ethics Committee at SYSUCC. Nude mice (BALB/c, male, 3-4 weeks old) were housed in specific pathogen-free animal facilities. The animal care was in accordance with the institution guidelines of Animal Ethics Committee at SYSUCC. *In vivo* tumor formation assay was performed by subcutaneously injecting cells into the right dorsal flank of mice. The mice were divided into 4 groups (n = 6 for each group) randomly: 1.5×10^6^ KYSE150 cells mixed with 5×10^5^ 3T3-PI16 cells (Group 1 and Group 3); 1.5×10^6^ KYSE150 cells mixed with 5×10^5^ 3T3-Vec cells (Group 2 and Group 4). Mice of Group 3 and 4 received an intraperitoneal injection of cisplatin (Hansoh Pharmaceutical Company, Jiangsu, China) (5 µg/g body weight) at day 5, day 10 and day 15 (Figure 4I). Tumor volume was measured every 5 days and calculated using the formula: volume = 0.5×L ×W^2^ (L, length; W, width). The mice were sacrificed on the 25^th^ day. Xenografts were isolated and fixed in 10% formalin.

For shRNA treatment experiments, BALB/c nude mice (male, 3-4 weeks old) were subcutaneously injected with 1.5×10^6^ KYSE150 cells mixed with 5×10^5^ 3T3-PI16 cells. On day 12, the mice were divided into two groups (n = 6 for each group) randomly. The mice received intratumoral injection of lentivirus-mediated shRNA-1 targeting PI16 (shRNA group) or control scrRNA (control group) (2×10^6^ TU/20 ul/mouse) every 5 days. Both groups received intraperitoneal injection of cisplatin (5 µg/g body weight) every 5 days. Tumor volume was measured and calculated as mentioned above. The mice were sacrificed on the 35^th^ day. Xenografts were isolated and fixed in 10% formalin.

For DHC treatment experiments, BALB/c nude mice (male, 3-4 weeks old) were subcutaneously injecting cells into the right dorsal flank of mice as described above. The mice were divided into 4 groups (n = 6 for each group) randomly. Group 1 and group 2 were injected with 1.5×10^6^ KYSE150 mixed with 5×10^5^ 3T3-Vec cells. Group 3 and group 4 were injected with 1.5×10^6^ KYSE150 mixed with 5×10^5^ 3T3-PI16 cells. On day 12, Animals were treated with intratumoral injection of Dehydrocorydaline chloride (DHC) (5 µg/g body weight) or DMSO control every 5 days. All groups received intraperitoneal injection of cisplatin (5 µg/g body weight) every 5 days. Tumor growth was observed as described above. The animals were sacrificed on the 32^nd^ day.

**Statistical analyses:**

The data are expressed as mean ± standard error of the mean (SEM). Statistical analyses of data were performed using SPSS (version 25.0) (Chicago, IL) and GraphPad Prism 7.0 software. A paired two-tailed *t*-test was performed to compare α-SMA expression in fibroblasts of ESCC tumor tissues and corresponding metastatic lymph node tissues. Pearson's or Spearman's tests were used to examine the correlation between two groups. Kaplan-Meier analysis with the log-rank test was used to analyze overall survival. Student's *t*-test was used to compare the differences between two groups. One-way analysis of variance tests was used to compare the differences among more than two groups. *P*<0.05 was considered statistically significant.

**Patients’ characteristics:**

| **patients** | **Patients’ characteristics** |
| --- | --- |
| patients with lymph node metastasis | Male: 29 cases; female: 5 cases;  Age distribution: 47-75 years old; Median age: 60 years old. |
| patients receiving platinum-based therapy | Male: 44 cases; female: 6 cases;  Age distribution: 40-74 years old; Median age: 54 years old. |
| TCGA-ESCA datasets (age<70 years at the time of surgery) | Male: 58 cases; female: 10 cases;  Age distribution: 36-69 years old; Median age: 54 years old. |

**Supplementary tables:**

Supplementary table 1. Univariate and multivariate analyses of prognostic factors in patients with ESCC

| Clinicopathological Features | Univariate Analysis | | | Multivariate Analysis | | |
| --- | --- | --- | --- | --- | --- | --- |
|  | HR | 95%CI | P | HR | 95%CI | P |
| Gender (Male vs Female) | 0.775 | 0.294-2.024 | 0.772 |  |  |  |
| Age (>60 vs <=60) | 1.13. | 0.569-2.244 | 0.726 |  |  |  |
| Tumor invasion | 3.006 | 1.030-8.778 | **0.044*** |  |  |  |
| Lymph node metastasis (1,2,3) | 1.614 | 0.952-2.737 | **0.076** |  |  |  |
| Distant metastasis | 3.617 | 0.445-29.426 | 0.229 |  |  |  |
| Clinical stage (II, III, IV) | 2.877 | 1.226-6.748 | **0.015*** | 2.995 | 1.219-7.358 | **0.017*** |
| PI16 upregulation in Fbs of LN | 2.206 | 1.059-4.595 | **0.035*** | 2.189 | 1.000-4.793 | **0.050*** |

HR, hazard ratio; CI, confidence interval.

ESCC patients (N=34)

**P*<0.05 represents statistically significant.

Supplementary table 2. Univariate and multivariate analyses of prognostic factors in ESCC patients of TCGA

| Clinicopathological Features | Univariate Analysis | | | Multivariate Analysis | | |
| --- | --- | --- | --- | --- | --- | --- |
|  | HR | 95%CI | P | HR | 95%CI | P |
| Gender (Male vs Female) | 8.691 | 1.129-66.913 | **0.038*** |  |  |  |
| Tumor invasion | 0.803 | 0.340-1.899 | 0.618 |  |  |  |
| Lymph node metastasis (0,1,2) | 4.269 | 1.883-9.681 | **0.001*** | 4.464 | 1.803-11.058 | **0.001*** |
| Distant metastasis | 3.719 | 1.204-11.483 | **0.022*** |  |  |  |
| Clinical stage | 3.523 | 1.275-9.738 | **0.015*** |  |  |  |
| Histologic grade | 1.134 | 0.562-2.289 | 0.726 |  |  |  |
| PI16 upregulation in mRNA | 2.797 | 1.031-7.589 | **0.043*** | 3.891 | 1.136-13.319 | **0.030*** |

HR, hazard ratio; CI, confidence interval.

ESCC patients: <70 years old on surgery. (N=68)

**P*<0.05 represents statistically significant.

Supplementary table 3: Univariate and multivariate analyses of prognostic factors for ESCC patients receiving platinum-based chemotherapy

| Clinicopathological Features | Univariate Analysis | | | Multivariate Analysis | | |
| --- | --- | --- | --- | --- | --- | --- |
|  | HR | 95%CI | P | HR | 95%CI | P |
| Gender (Male vs Female) | 4.594 | 0.623-33.884 | **0.135** |  |  |  |
| Age (>60 vs <=60) | 1.195 | 0.481-2.965 | 0.701 |  |  |  |
| Tumor invasion | 4.726 | 1.109-20.140 | **0.036*** | 4.432 | 1.035-18.987 | **0.045*** |
| Lymph node metastasis (0,1,2,3) | 1.596 | 1.053-2.419 | **0.027*** |  |  |  |
| Distant metastasis | 1.342 | 0.180-9.980 | 0.774 |  |  |  |
| Clinical stage | 2.150 | 0.739-6.254 | 0.160 |  |  |  |
| PI16 upregulation in interstitial | 3.152 | 1.425-6.973 | **0.005*** | 2.527 | 1.129-5.658 | **0.024*** |

HR, hazard ratio; CI, confidence interval.

ESCC patients: receiving post-operation platinum-based chemotherapy. (N=50)

**P*<0.05 represents statisticallysignificant.

**Supplementary figures and figure legends:**


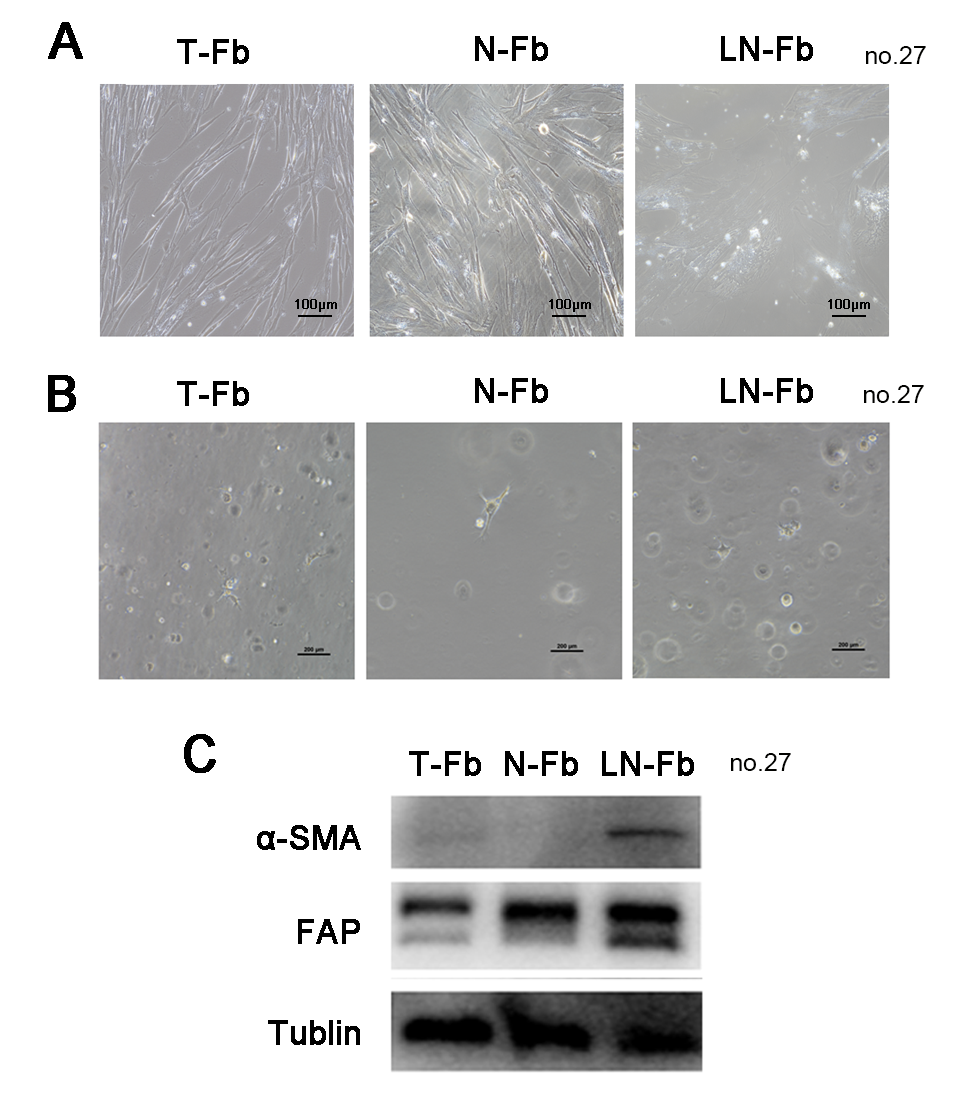


**Supplementary figure 1: Characteristics of fibroblasts derived from tumor tissues, nontumor tissues and metastatic lymph nodes.**

A) Representative images of fibroblasts derived from tumor tissues (T-Fbs), nontumor tissues (N-Fbs) and metastatic lymph nodes (LN-Fbs) cultured on plastic. B) Bright field images of T-Fbs, N-Fbs and LN-Fbs cultured in Matrigel. C) Western blotting results for α-SMA and FAP in T-Fbs, N-Fbs and LN-Fbs cultured in Matrigel. β-tubulin was used as a loading control.

**
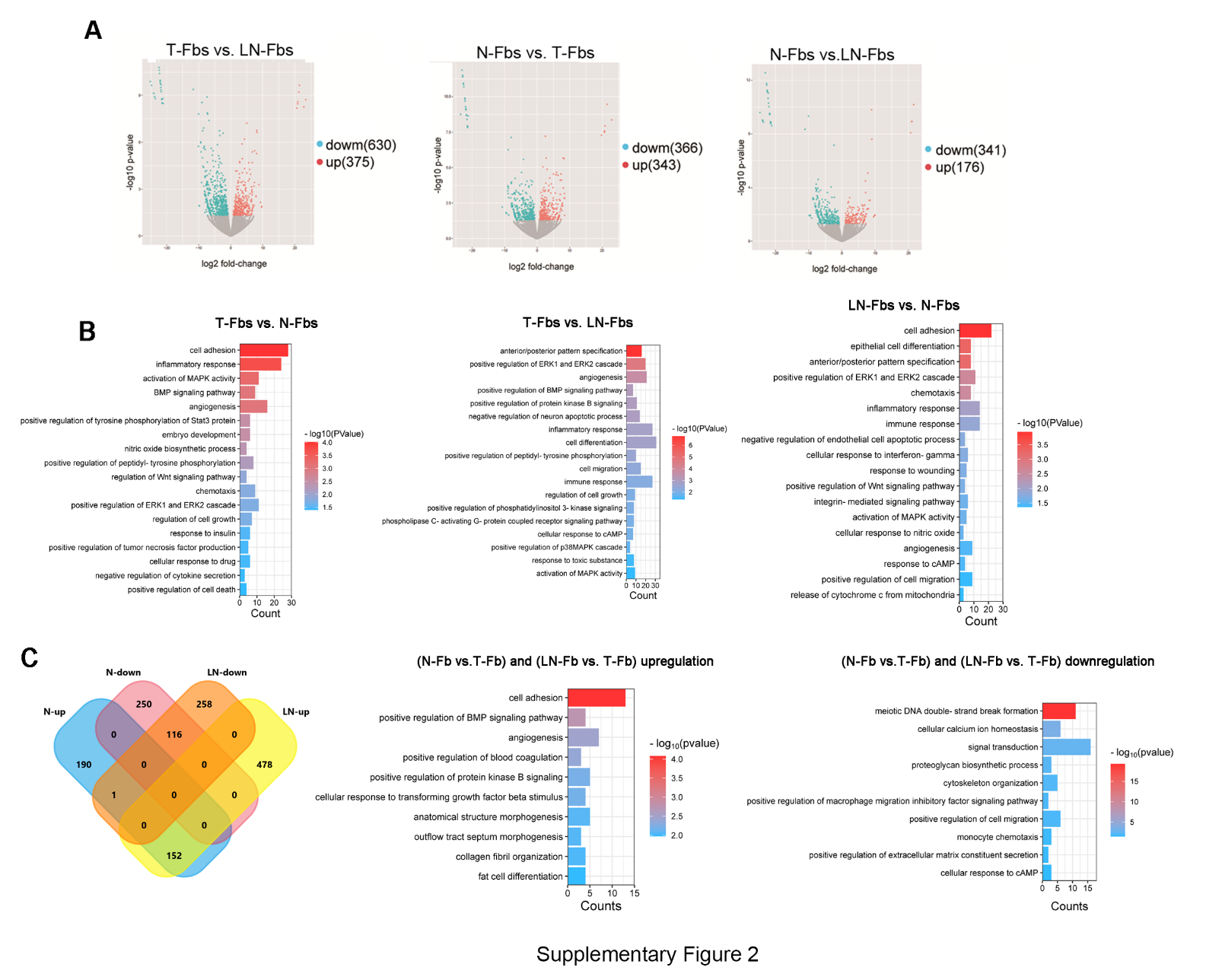
**

**Supplementary figure 2: Transcriptomic profiling of fibroblasts derived from tumor tissues, nontumor tissues and metastatic lymph nodes.**

1. Volcano plots of upregulated or downregulated genes in T-Fbs vs. LN-Fbs, N-Fbs vs. T-Fbs, and N-Fbs vs. LN-Fbs. B) GO biological pathway analysis of RNA-seq results of T-Fbs vs. N-Fbs, T-Fbs vs. LN-Fbs, and LN-Fbs vs. N-Fbs. C) Venn diagram of the upregulated or downregulated genes in N-Fbs vs. T-Fbs and LN-Fbs vs. T-Fbs, and top 10 most significant Gene Ontology (GO) terms identified by GO biological pathway analysis of the common upregulated or downregulated genes in the two comparisons.


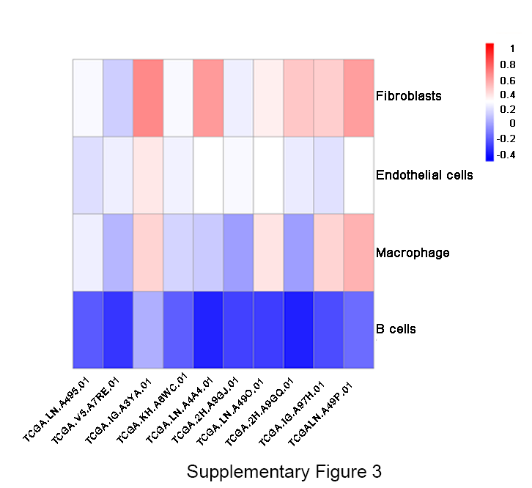


**Supplementary figure 3: Tirosh score generated by xCell and cell-type enrichment analysis results of transcriptome files of 10 cases of TCGA-ESCA.**

**
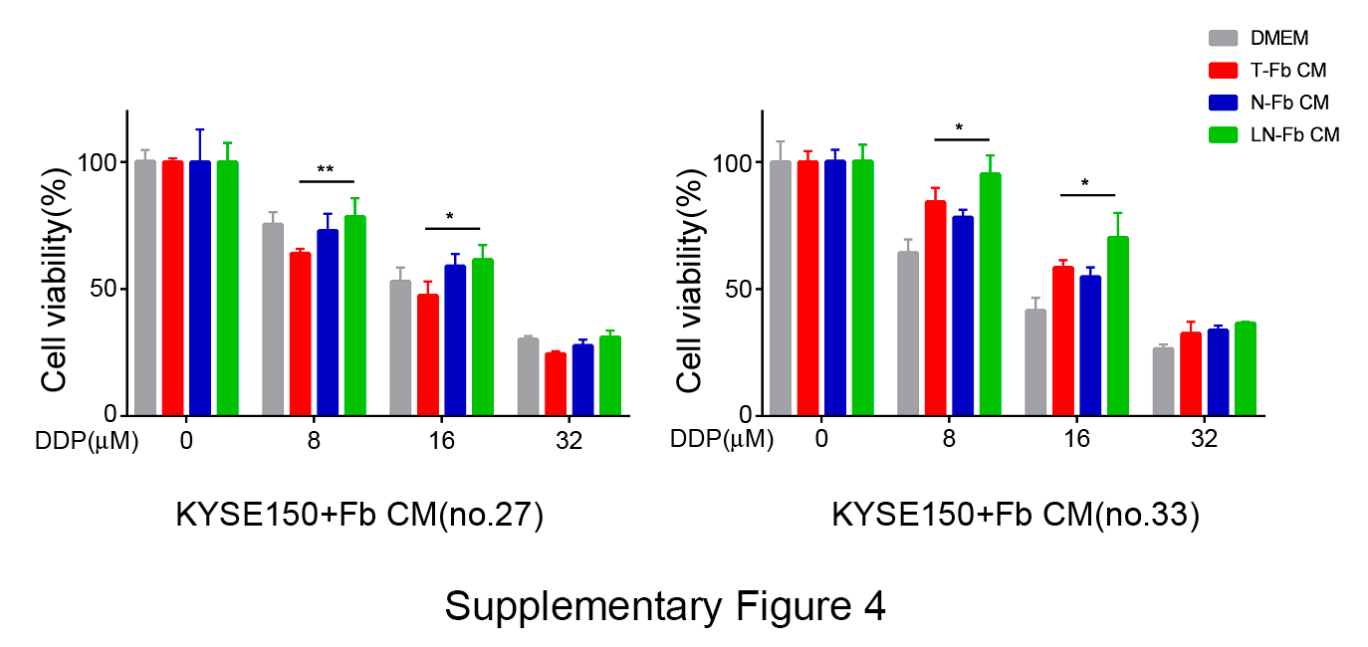
**

**Supplementary figure 4: Coculturing with LN-Fbs induces resistance to cisplatin in ESCC cells.**

Cell viability assays of KYSE150 cells cocultured with CM from T-Fbs, N-Fbs and LN-Fbs from different ESCC patients (no. 27 and no. 33). Then, the cells were treated with different concentrations of DDP (*, *P*<0.05; **, *P*<0.01).


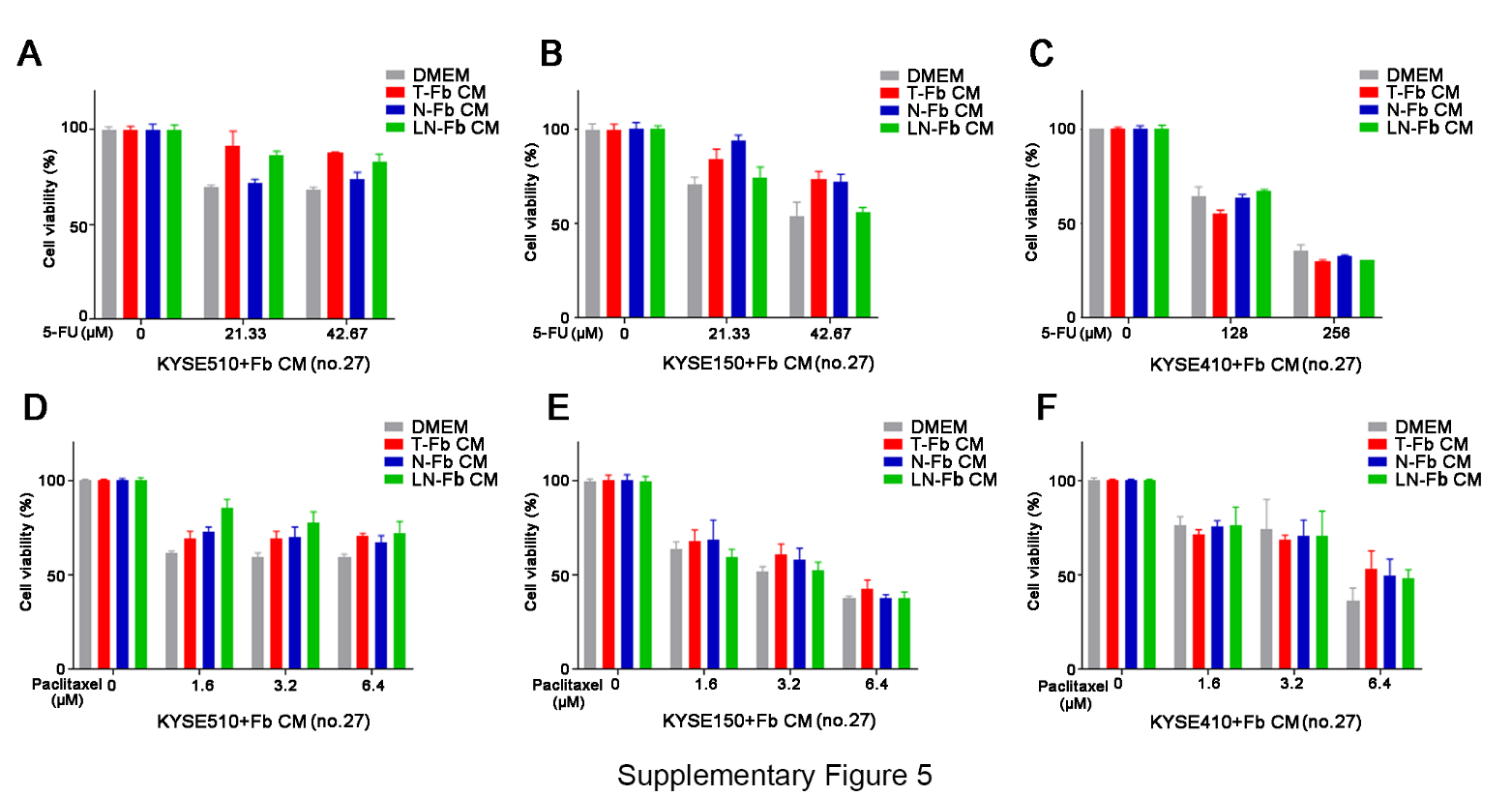


**Supplementary figure 5. Coculturing with fibroblasts does not induce resistance to fluorouracil or paclitaxel in ESCC cells.**

A, B, C) Cell viability assay of KYSE510 (A), KYSE150 (B) and KYSE410 (C) cells cocultured with CM from T-Fbs, N-Fbs and LN-Fbs of ESCC patients (no. 27). ESCC cells were treated with different concentrations of fluorouracil (5-Fu). D, E, F) Cell viability assay of KYSE510 (D), KYSE150 (E) and KYSE410 (F) cells cocultured with CM from T-Fbs, N-Fbs and LN-Fbs of ESCC patients (no. 27). ESCC cells were treated with different concentrations of paclitaxel.


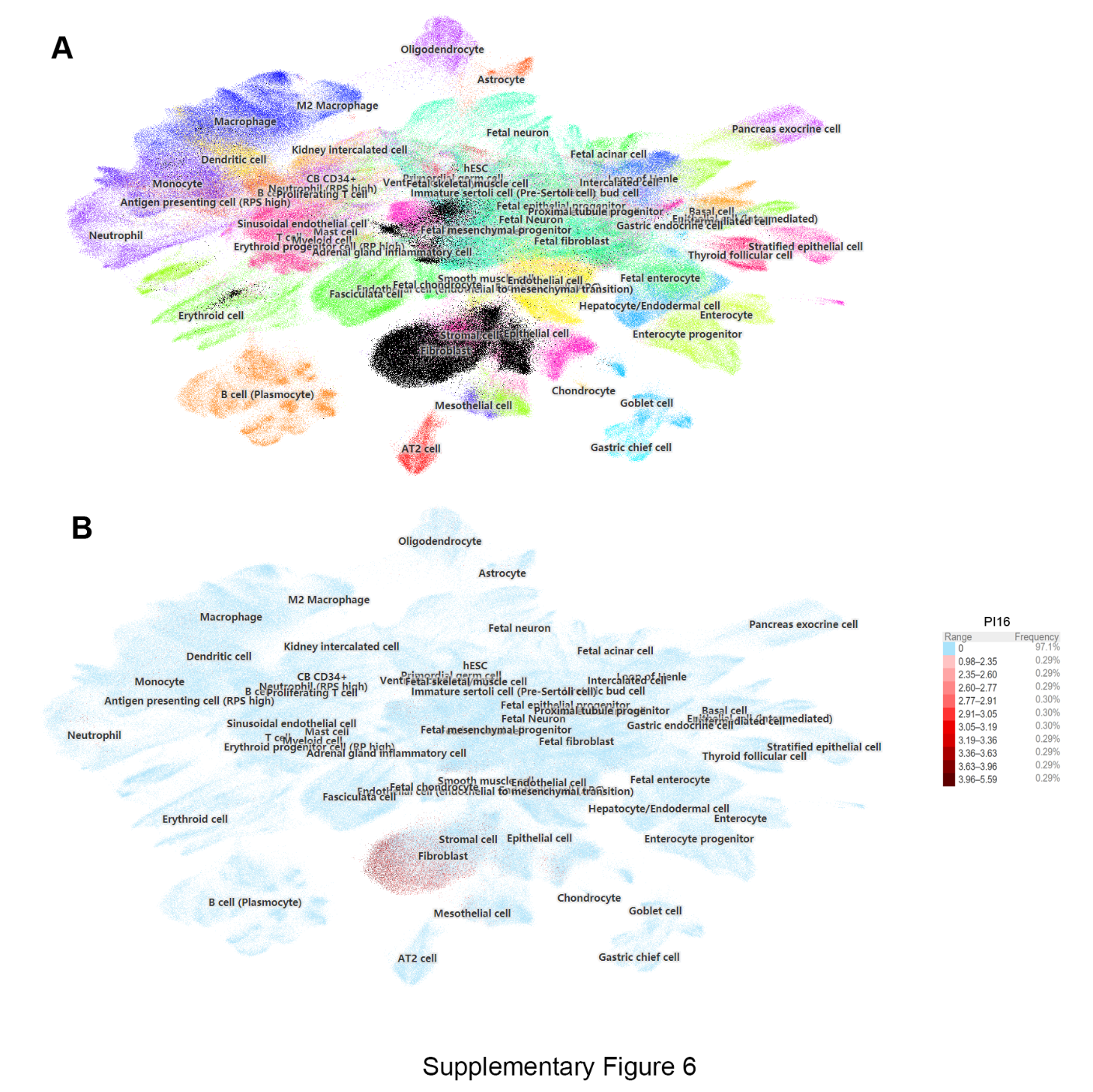


**Supplementary figure 6: Human cellular landscape and PI16 expression**.

1. Unsupervised clustering of viable cells from by single-cell mRNA sequencing. Different cell types are color-coded (Fibroblasts, black). B) PI16 expression in human cellular landscape (A). Data were extracted from GEO (GSE134355) or Bioproject (PRJNA554845).


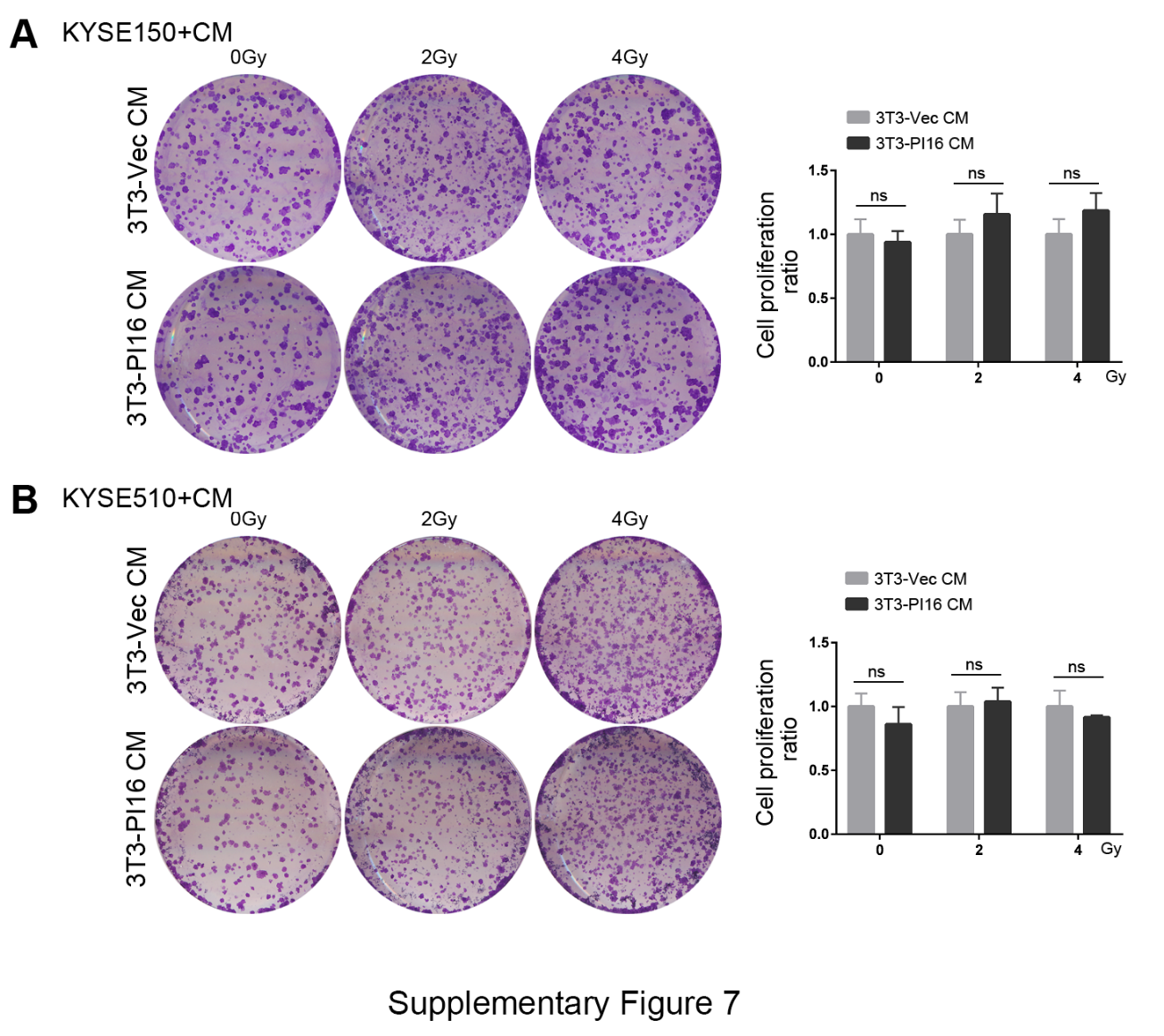


**Supplementary figure 7: Coculturing with 3T3-PI16 CM has no effect on ESCC cell radiation resistance.**

A, B) KYSE150 (A) or KYSE510 (B) cells cocultured with CM of 3T3-PI16 cells or 3T3-Vec cells were treated with different doses of radiation. Cell proliferation ratio was summarized (*right*) (ns, not significant).


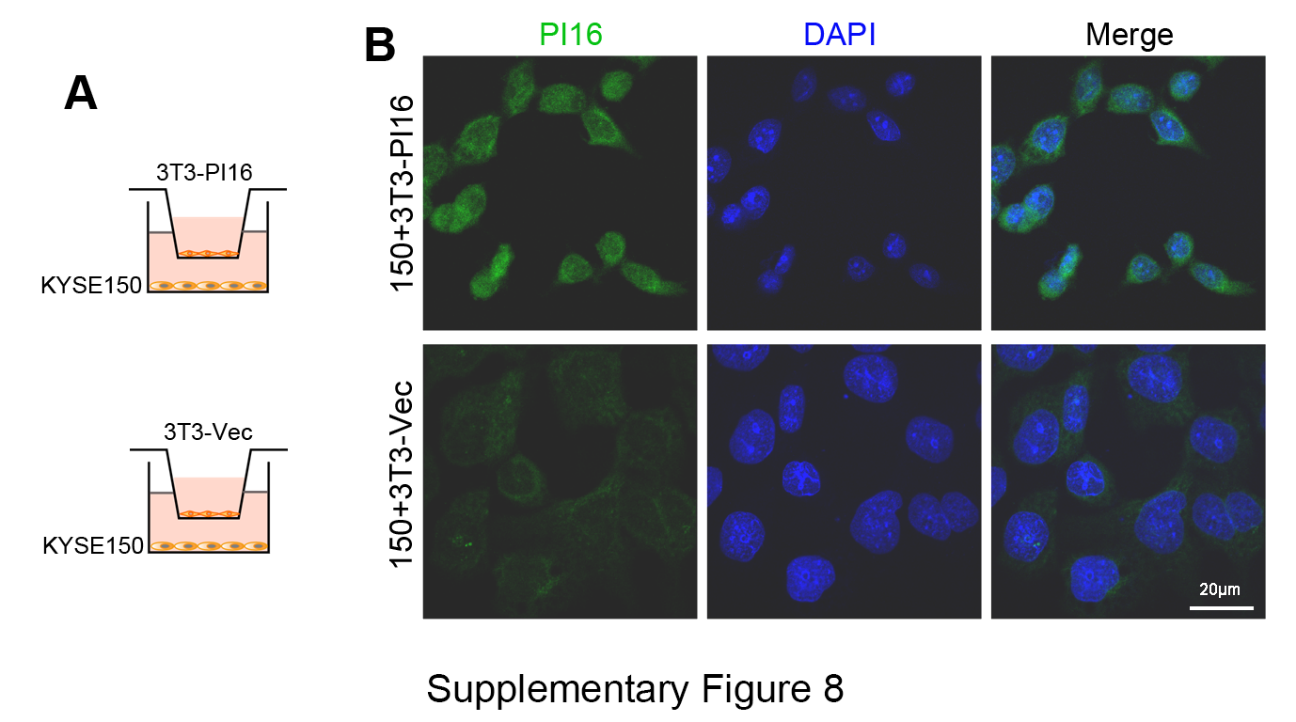


**Supplementary figure 8: Immunofluorescence staining of PI16 in KYSE150.**

1. Schematic of KYSE150 cells cocultured with NIH3T3 derivative cells (3T3-PI16 and 3T3-Vec). B) Representative images of immunofluorescence staining of PI16 in KYSE150 cells cocultured with 3T3-PI16 or 3T3-Vec cells. PI16 (green); DAPI (blue, counterstain).


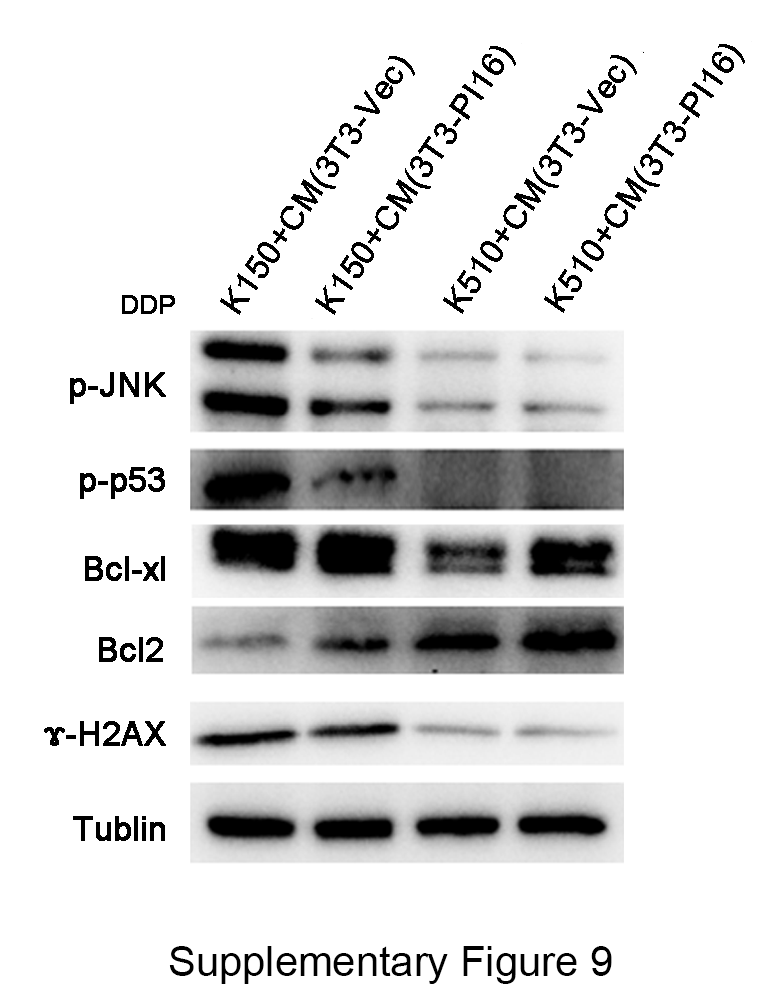


**Supplementary figure 9: Western blotting results of ESCC cells treated with DDP.**

Western blotting results of p-JNK, p-P53 (ser15), Bcl-xL, Bcl2 and γ-H2AX in KYSE150 or KYSE510 cells cultured with CM of 3T3-PI16 or 3T3-Vec in the presence of cisplatin.

(For KYSE150 cells: 80 uM DDP, 6 h; For KYSE510 cells, 20 uM DDP, 6 h )


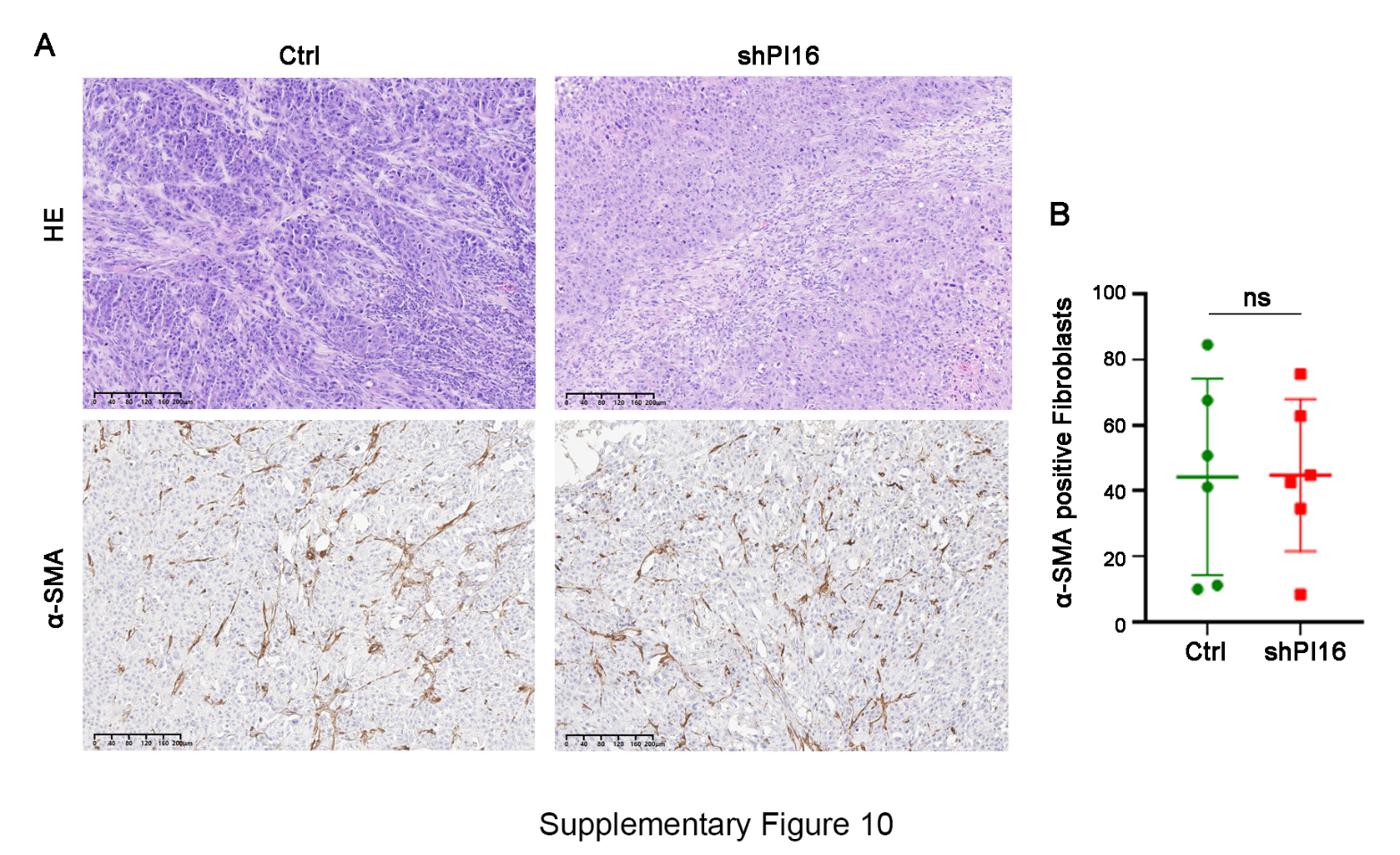


**Supplementary figure 10: The amount of fibroblasts in the shPI16 treatment animal experiments.**

1. Representative images of HE staining and IHC of α-SMA staining in xenografts sections of the shPI16-treated animals compared to the control group. B) The summary of cell counts of α-SMA positive fibroblasts in xenografts. (ns, not significant)


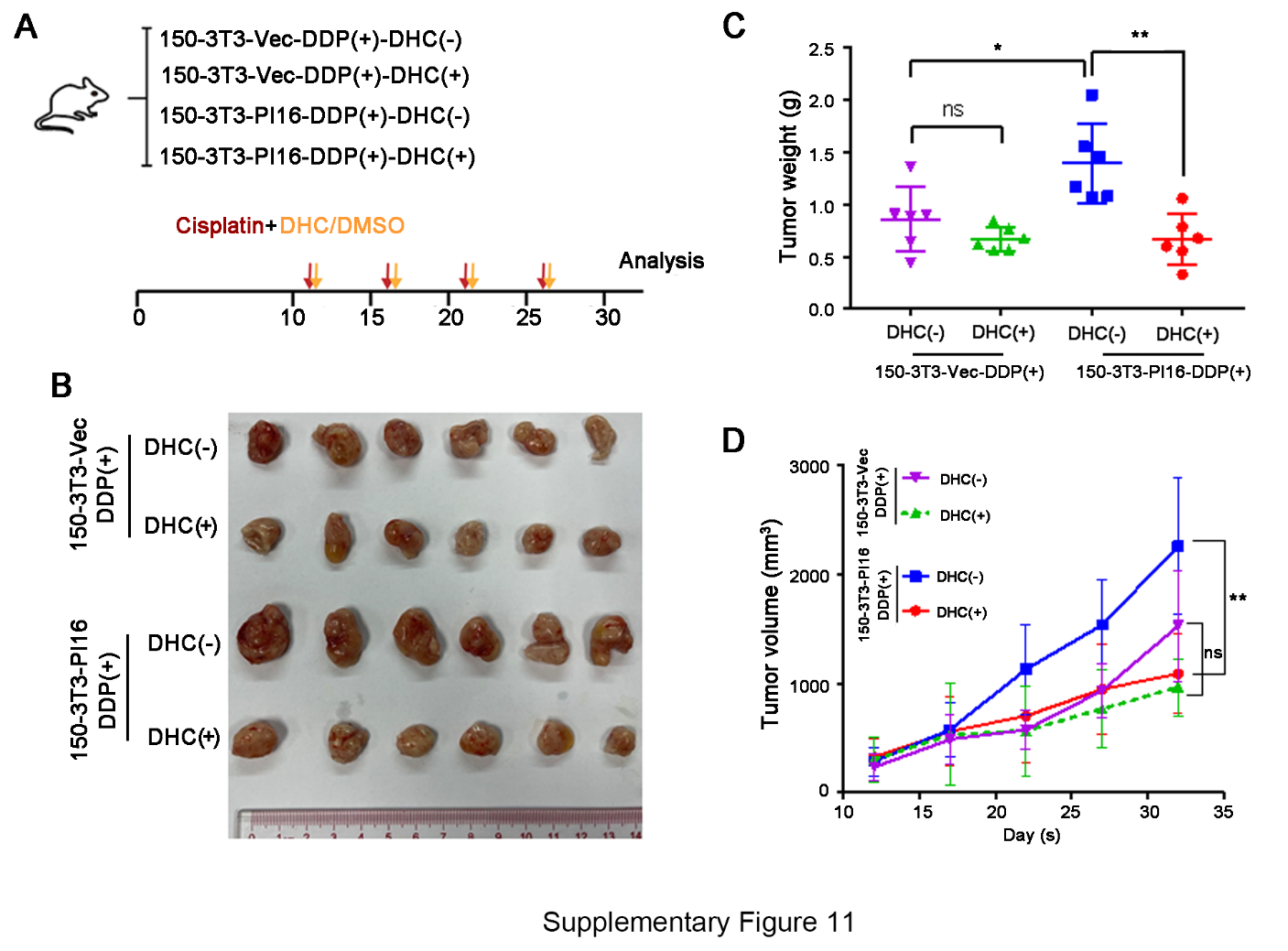


**Supplementary figure 11: p38 MAPK activation enhances sensitivity of ESCC cells to cisplatin in *in vivo* assay.**

A) Graphical scheme describing the workflow of animal experiments. B-D) The image (B), tumor weight (C) and tumor growth curve (D) of xenografts formed in nude mice receiving the combination of DDP with Dehydrocorydaline chloride (DHC) or DMSO control. (ns: not significant; *, *P*<0.05; **, *P*<0.01)
